# Supplementary material for: Understanding seed-mediated growth of gold nanoclusters at molecular level
Source: Nat Commun. 2017 Oct 13;8:927. doi: 10.1038/s41467-017-00970-1 (PMC5640603; doi:10.1038/s41467-017-00970-1)
Supplement: Supplementary file 1 — Supplementary Information [file 41467_2017_970_MOESM1_ESM.pdf]

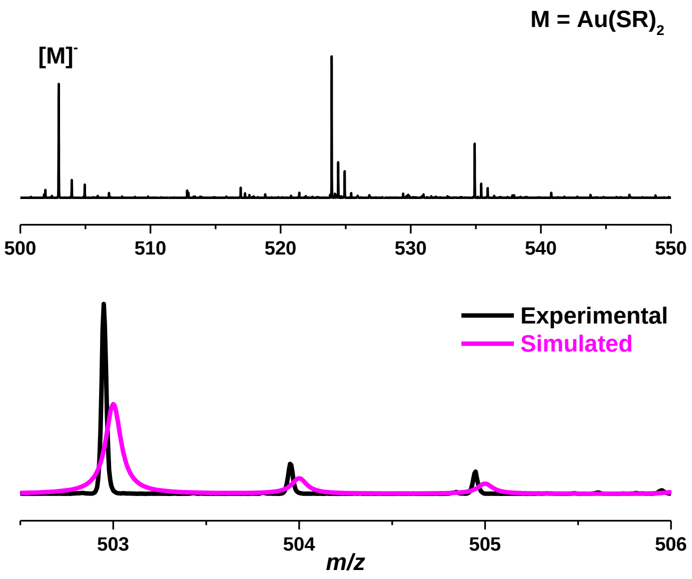

Supplementary Figure 1. Electrospray ionization mass spectrum (top) and isotope patterns (bottom) of $[Au(SR)_2]^-$ (species **1**) obtained from 0 min sample.

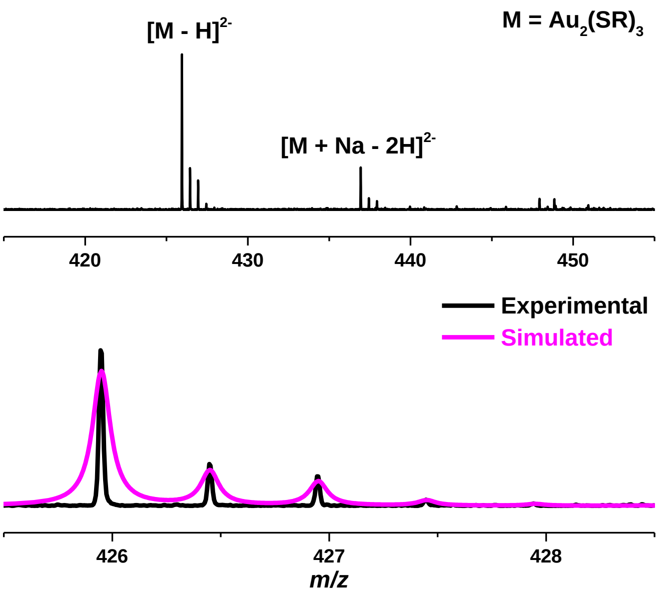

Supplementary Figure 2. Electrospray ionization mass spectrum (top) and isotope patterns (bottom) of $[Au_2(SR)_3 -H]^{2-}$ (species **2**) obtained from 0 min sample.

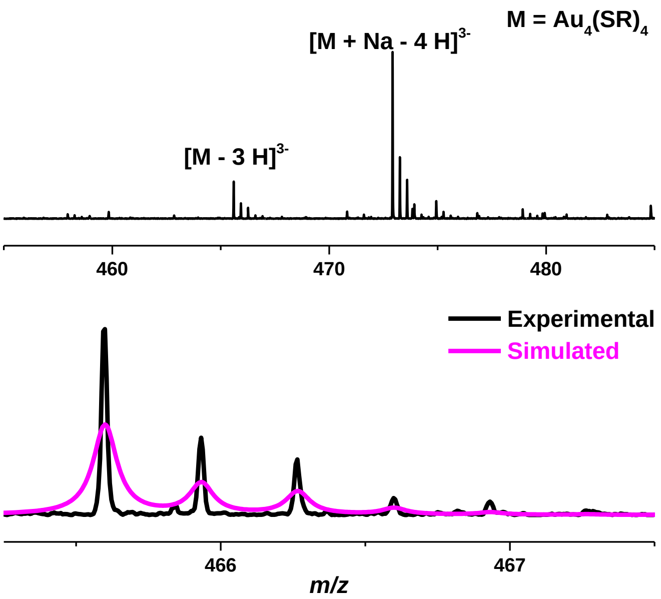

Supplementary Figure 3. Electrospray ionization mass spectrum (top) and isotope patterns (bottom) of $[Au_4(SR)_4 – 3\ H]^{3-}$ (species **3**) obtained from 0 min sample.

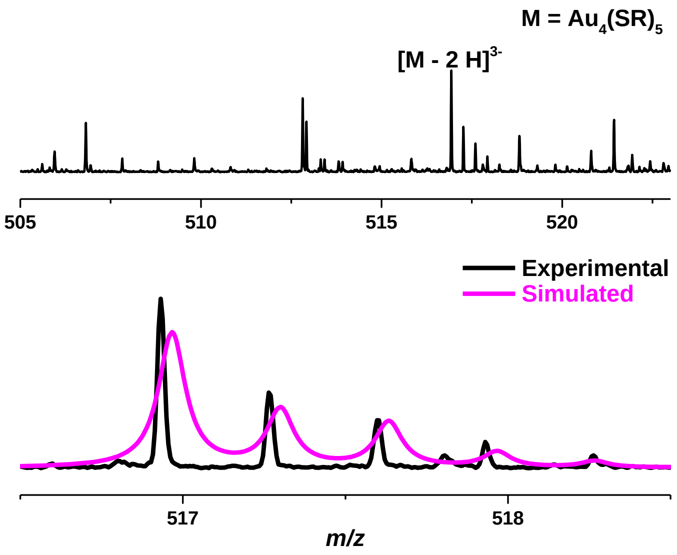

Supplementary Figure 4. Electrospray ionization mass spectrum (top) and isotope patterns (bottom) of $[Au_4(SR)_5 – 2\ H]^{3-}$ (species **4**) obtained from 0 min sample.

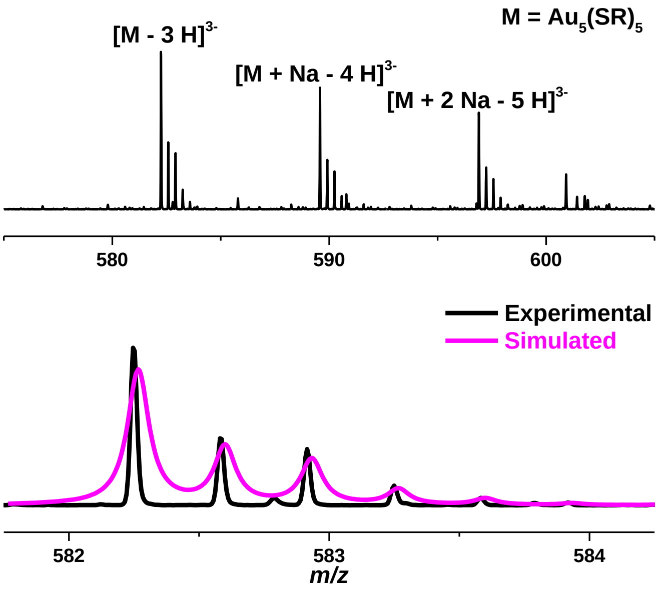

Supplementary Figure 5. Electrospray ionization mass spectrum (top) and isotope patterns (bottom) of $[Au_5(SR)_5 - 3\ H]^{3-}$ (species **5**) obtained from 0 min sample.

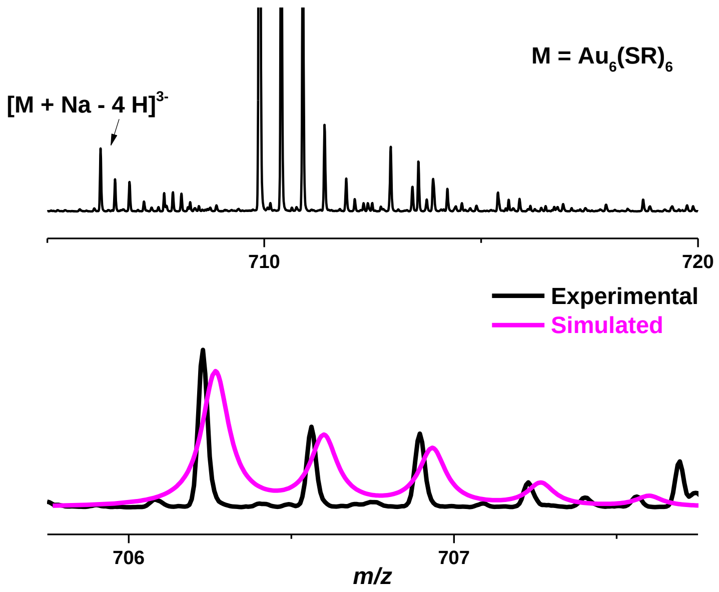

Supplementary Figure 6. Electrospray ionization mass spectrum (top) and isotope patterns (bottom) of $[Au_6(SR)_6 + Na – 4 H]^{3-}$ (species **6**) obtained from 0 min sample.

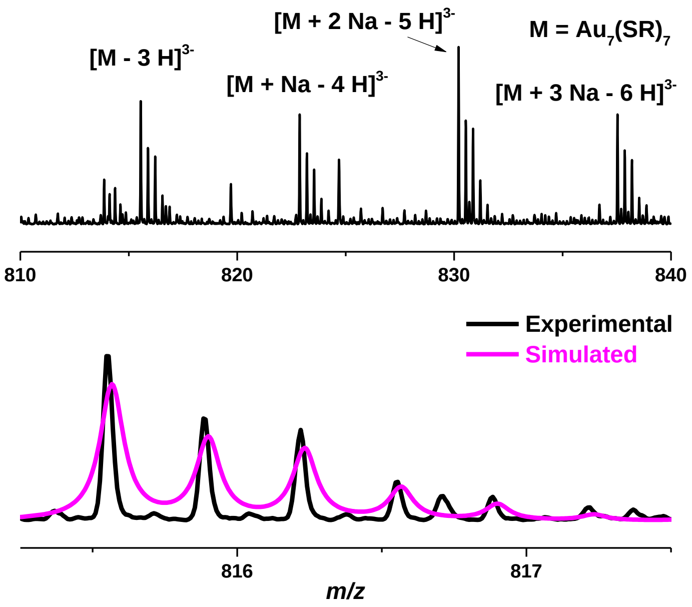

Supplementary Figure 7. Electrospray ionization mass spectrum (top) and isotope patterns (bottom) of $[Au_7(SR)_7 - 3\ H]^{3-}$ (species **7**) obtained from 0 min sample.

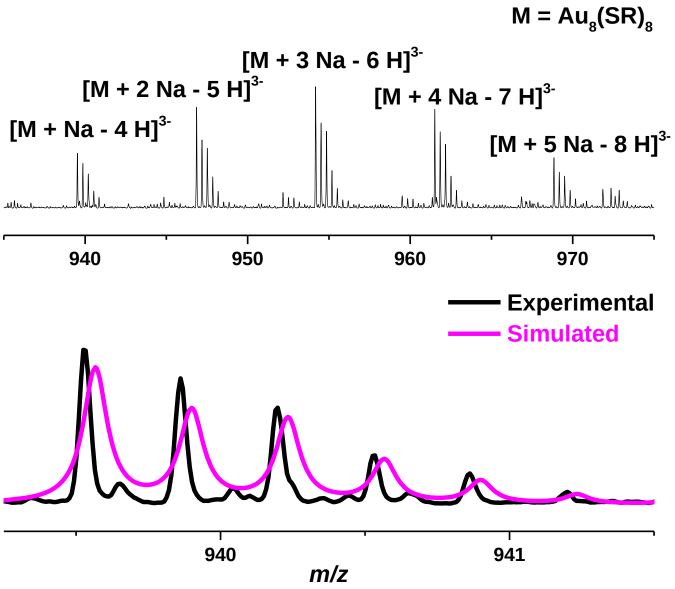

Supplementary Figure 8. Electrospray ionization mass spectrum (top) and isotope patterns (bottom) of $[Au_8(SR)_8 + Na - 4\ H]^{3-}$ (species **8**) obtained from 0 min sample.

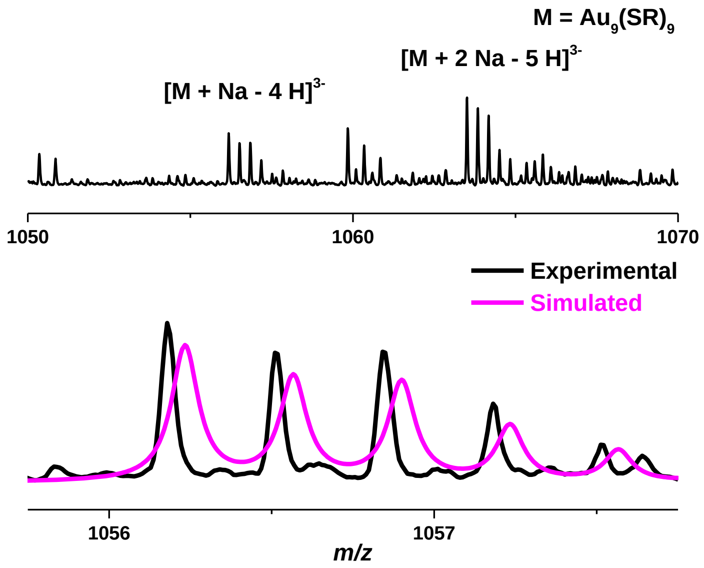

Supplementary Figure 9. Electrospray ionization mass spectrum (top) and isotope patterns (bottom) of $[Au_9(SR)_9 + Na – 4 H]^{3-}$ (species **9**) obtained from 0 min sample.

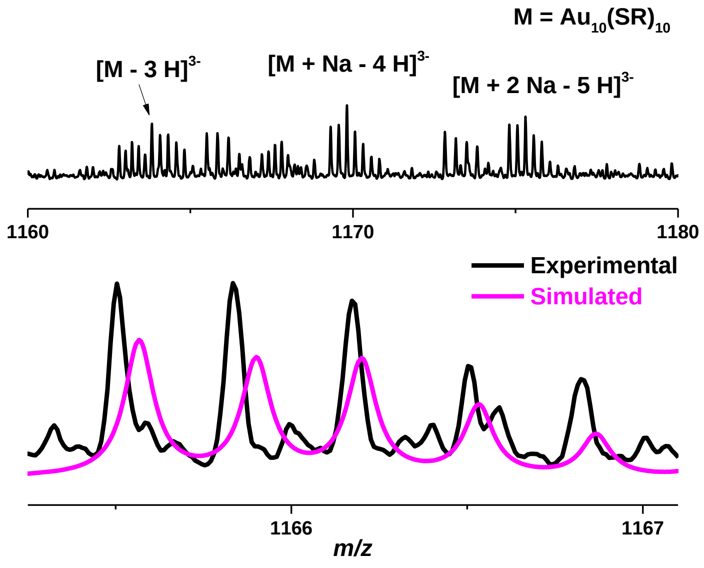

Supplementary Figure 10. Electrospray ionization mass spectrum (top) and isotope patterns (bottom) of $[Au_{10}(SR)_{10} - 3\ H]^{3-}$ (species **10**) obtained from 0 min sample.

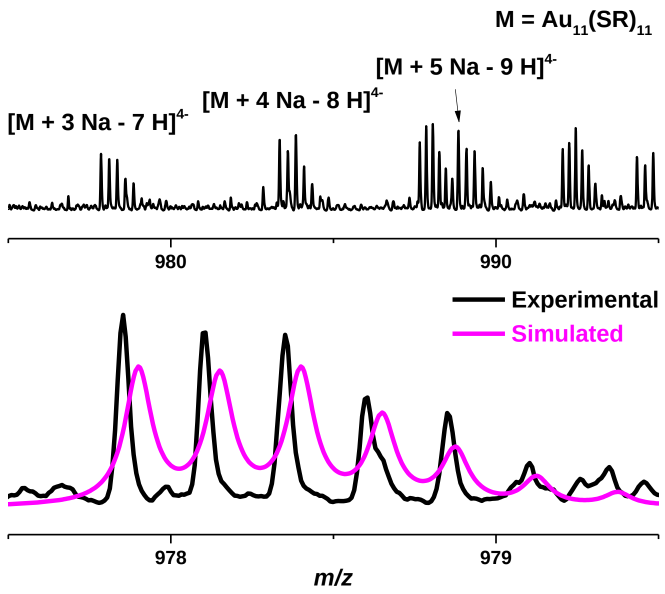

Supplementary Figure 11. Electrospray ionization mass spectrum (top) and isotope patterns (bottom) of $[Au_{11}(SR)_{11} + 3\ Na - 7\ H]^{4-}$ (species **11**) obtained from 0 min sample.

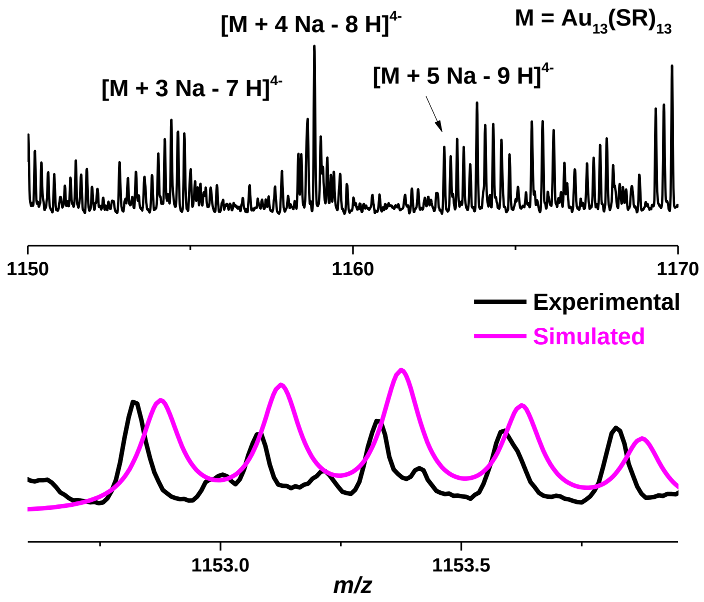

Supplementary Figure 12. Electrospray ionization mass spectrum (top) and isotope patterns (bottom) of $[Au_{13}(SR)_{13} + 3\ Na - 7\ H]^{4-}$ (species **12**) obtained from 0 min sample.

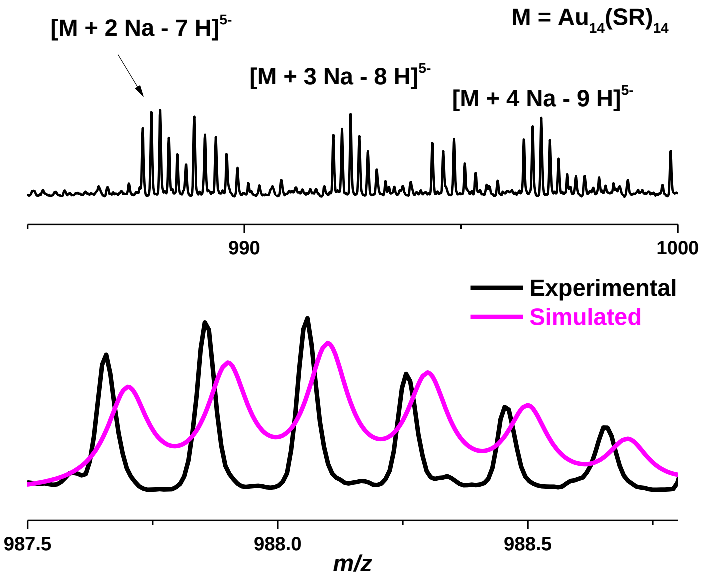

Supplementary Figure 13. Electrospray ionization mass spectrum (top) and isotope patterns (bottom) of $[Au_{14}(SR)_{14} + 2\ Na - 7\ H]^{5-}$ (species **13**) obtained from 0 min sample.

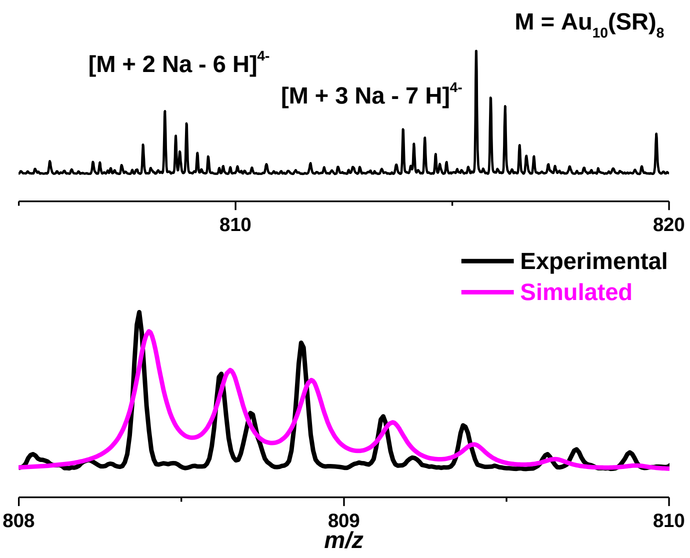

Supplementary Figure 14. Electrospray ionization mass spectrum (top) and isotope patterns (bottom) of $[Au_{10}(SR)_8 + 2\ Na - 6\ H]^{4-}$ (species **14**) obtained from 0 min sample.

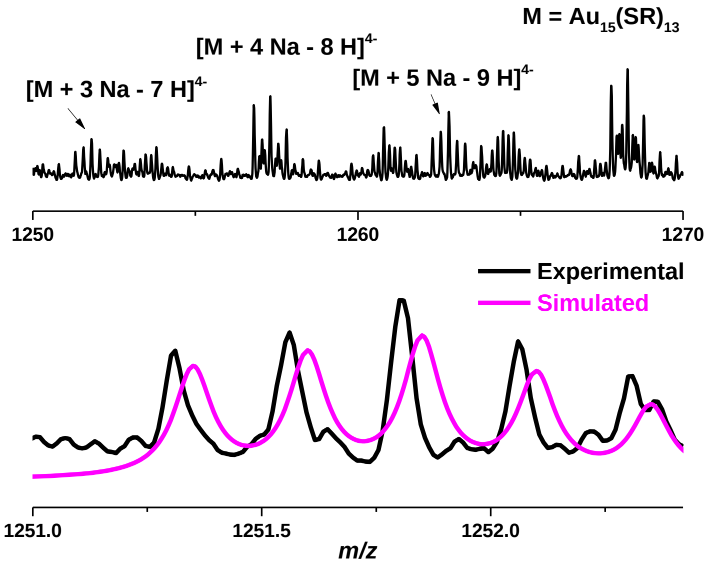

Supplementary Figure 15. Electrospray ionization mass spectrum (top) and isotope patterns (bottom) of $[Au_{15}(SR)_{13} + 3\ Na - 7\ H]^{4-}$ (species **15**) obtained from 0 min sample.

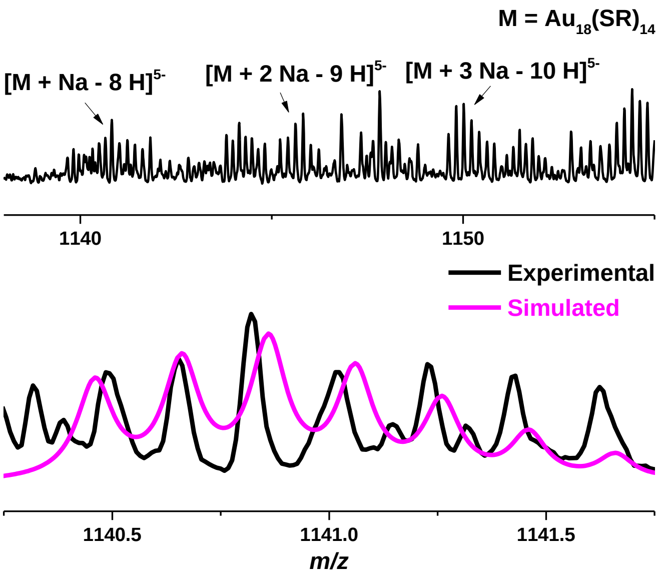

Supplementary Figure 16. Electrospray ionization mass spectrum (top) and isotope patterns (bottom) of $[Au_{18}(SR)_{14} + Na - 8\ H]^{5-}$ (species **16**) obtained from 0 min sample.

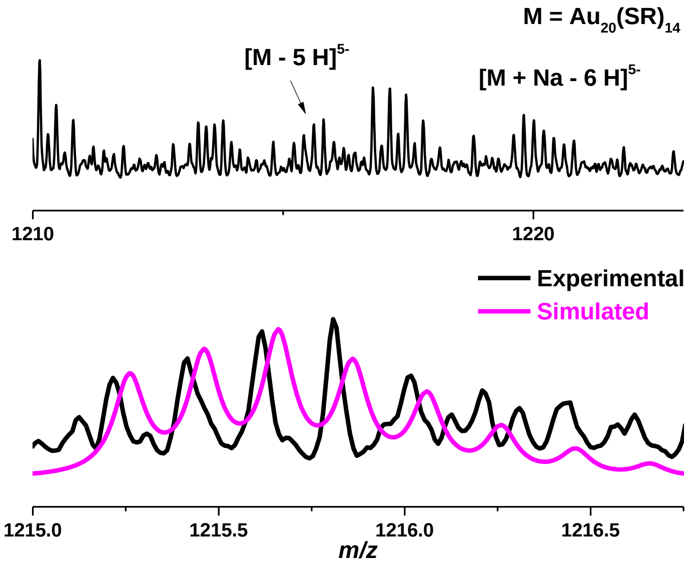

Supplementary Figure 17. Electrospray ionization mass spectrum (top) and isotope patterns (bottom) of $[Au_{20}(SR)_{14} - 5\ H]^{5-}$ (species **17**) obtained from 0 min sample.

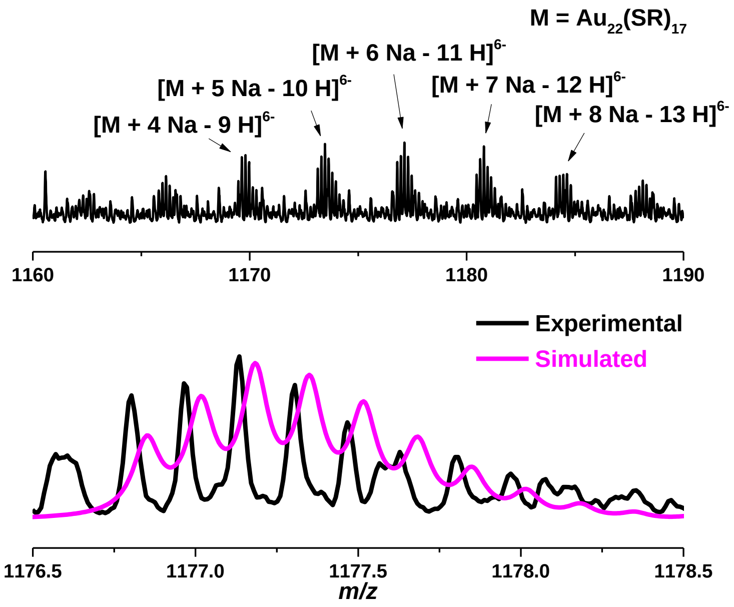

Supplementary Figure 18. Electrospray ionization mass spectrum (top) and isotope patterns (bottom) of $[Au_{22}(SR)_{17} + 6\ Na - 11\ H]^{6-}$ (species **18**) obtained from 0 min sample.

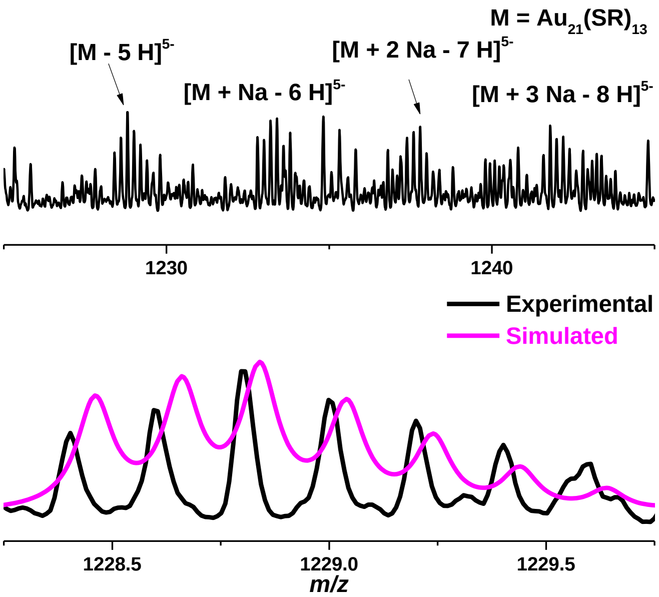

Supplementary Figure 19. Electrospray ionization mass spectrum (top) and isotope patterns (bottom) of $[Au_{21}(SR)_{13} - 5\ H]^{5-}$ (species **19**) obtained from 0 min sample.

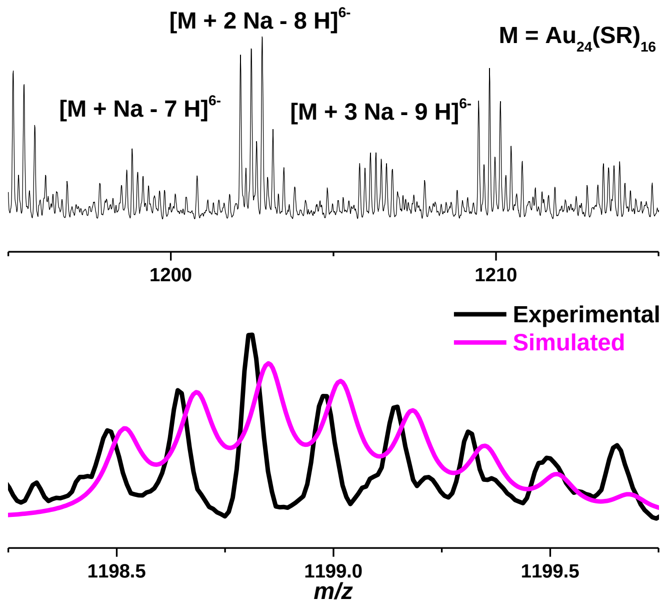

Supplementary Figure 20. Electrospray ionization mass spectrum (top) and isotope patterns (bottom) of $[Au_{24}(SR)_{16} + Na - 7\ H]^{6-}$ (species **20**) obtained from 0 min sample.

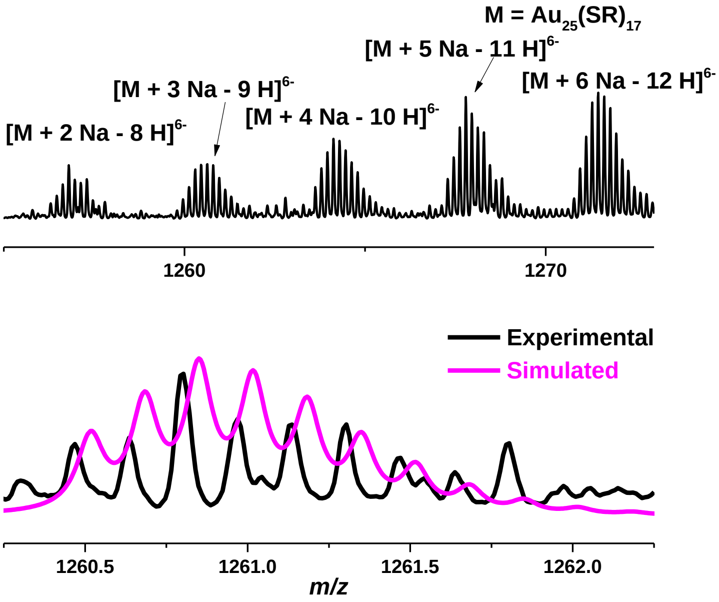

Supplementary Figure 21. Electrospray ionization mass spectrum (top) and isotope patterns (bottom) of $[Au_{25}(SR)_{17} + 2\ Na - 8\ H]^{6-}$ (species **21**) obtained from 10 min sample.

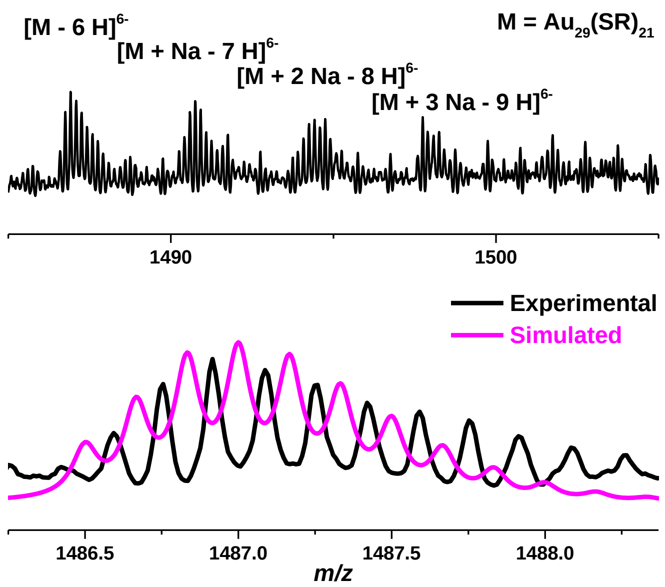

Supplementary Figure 22. Electrospray ionization mass spectrum (top) and isotope patterns (bottom) of $[Au_{29}(SR)_{21} – 6\ H]^{6-}$ (species **23**) obtained from 10 min sample.

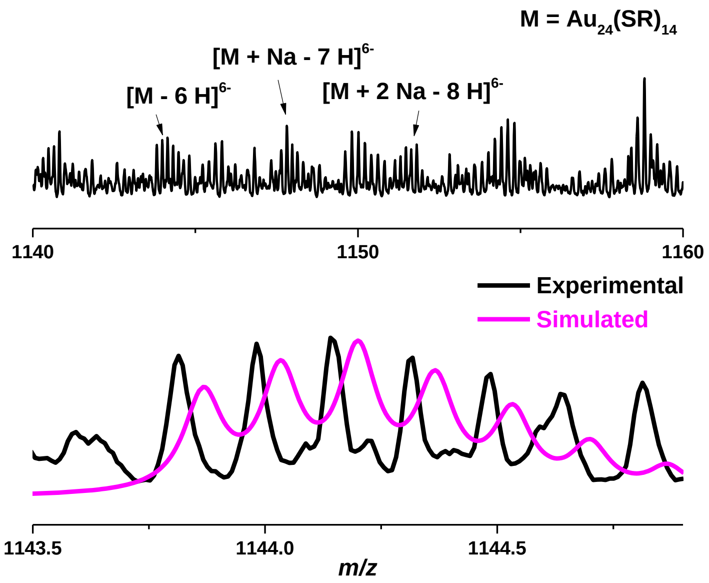

Supplementary Figure 23. Electrospray ionization mass spectrum (top) and isotope patterns (bottom) of $[Au_{24}(SR)_{14} – 6\ H]^{6-}$ (species **24**) obtained from 10 min sample.

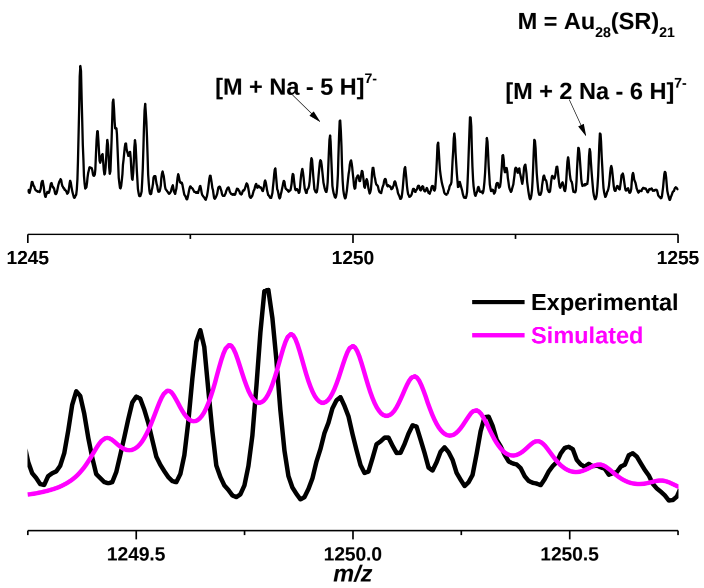

Supplementary Figure 24. Electrospray ionization mass spectrum (top) and isotope patterns (bottom) of $[Au_{28}(SR)_{21} + Na - 5\ H]^{7-}$ (species **25**) obtained from 10 min sample.

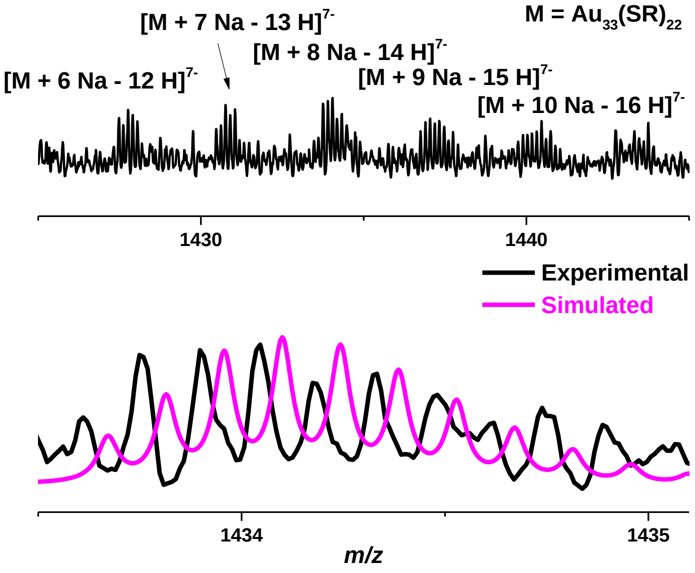

Supplementary Figure 25. Electrospray ionization mass spectrum (top) and isotope patterns (bottom) of $[Au_{33}(SR)_{22} + 8\ Na - 14\ H]^{7-}$ (species **26**) obtained from 1 h sample.

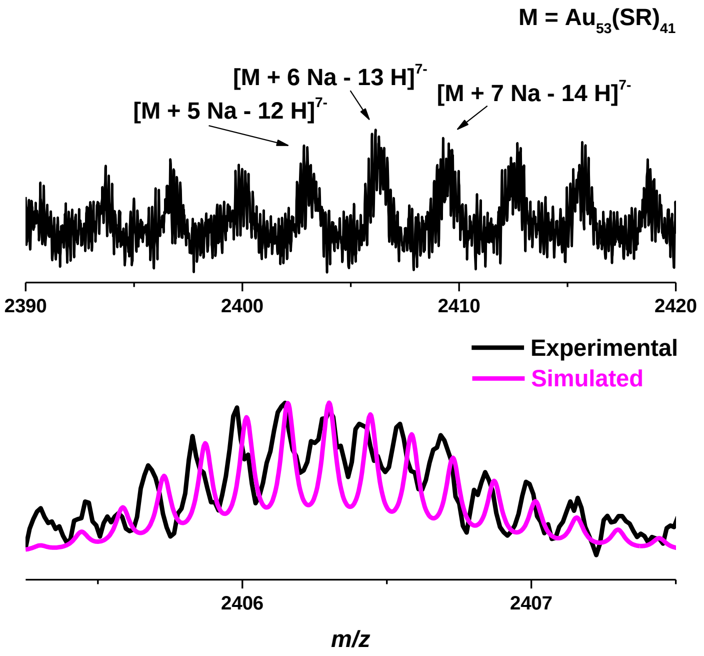

Supplementary Figure 26. Electrospray ionization mass spectrum (top) and isotope patterns (bottom) of $[Au_{53}(SR)_{41} + 6\ Na - 13\ H]^{7-}$ (species **27**) obtained from 24 h sample.

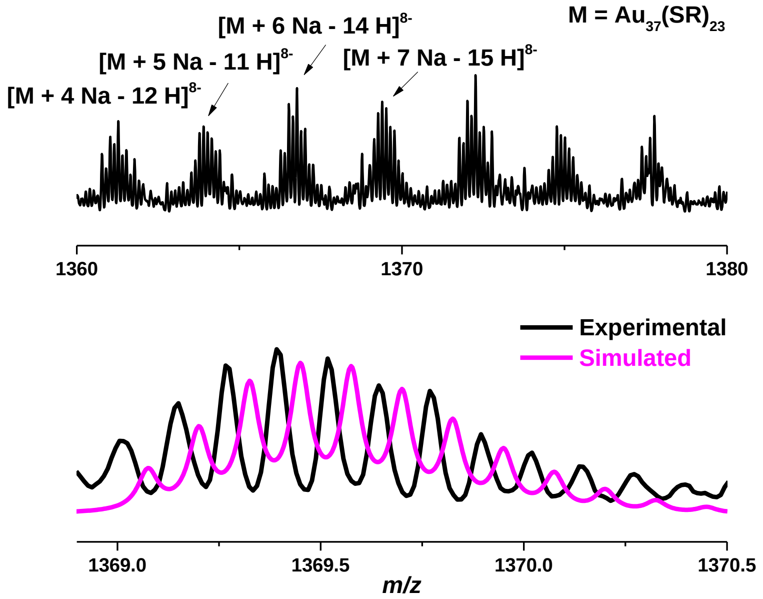

Supplementary Figure 27. Electrospray ionization mass spectrum (top) and isotope patterns (bottom) of $[Au_{37}(SR)_{23} + 7\ Na – 15\ H]^{8-}$ (species **28**) obtained from 10 min sample.

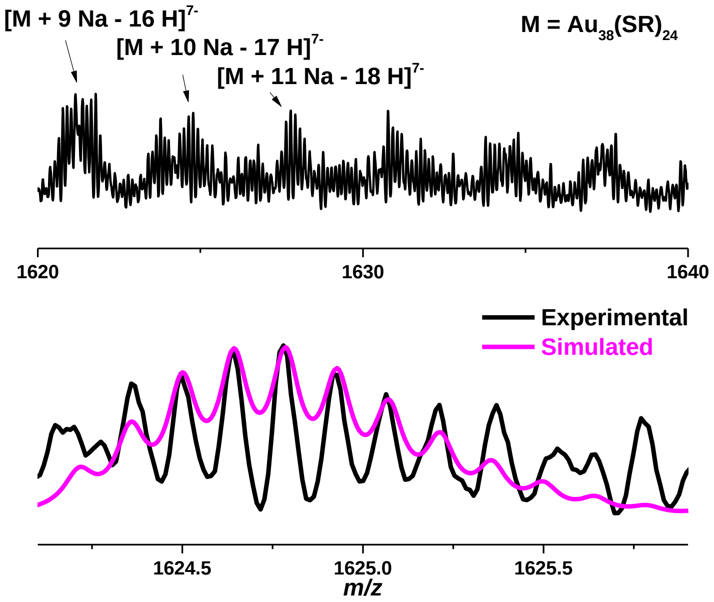

Supplementary Figure 28. Electrospray ionization mass spectrum (top) and isotope patterns (bottom) of $[Au_{38}(SR)_{24} + 10\ Na - 17\ H]^{7-}$ (species **29**) obtained from 4 h sample.

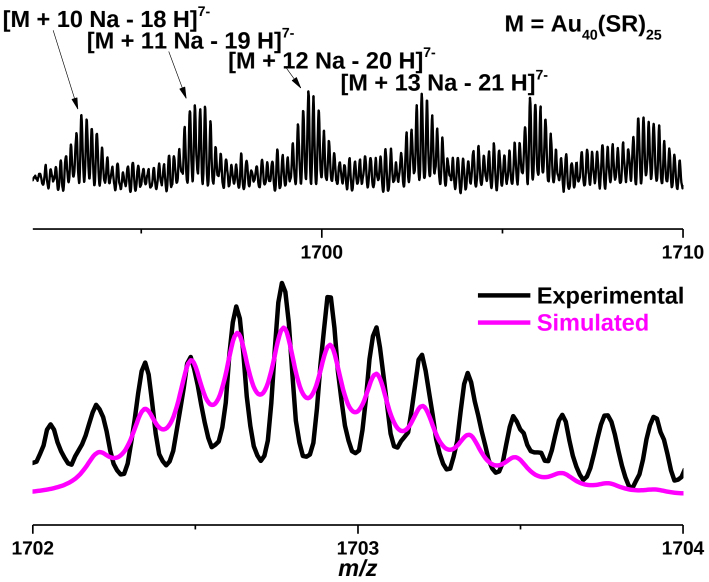

Supplementary Figure 29. Electrospray ionization mass spectrum (top) and isotope patterns (bottom) of $[Au_{40}(SR)_{25} + 10\ Na - 18\ H]^{7-}$ (species **30**) obtained from 10 h sample.

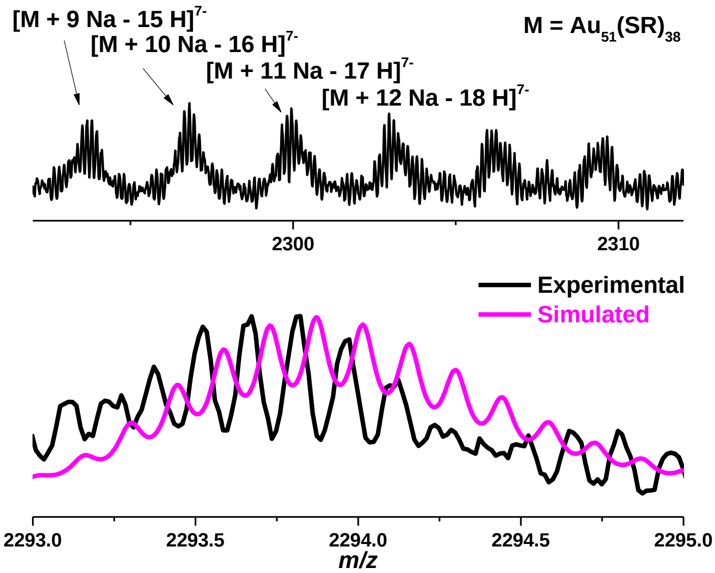

Supplementary Figure 30. Electrospray ionization mass spectrum (top) and isotope patterns (bottom) of $[Au_{51}(SR)_{38} + 9\ Na - 15\ H]^{7-}$ (species **31**) obtained from 12 h sample.

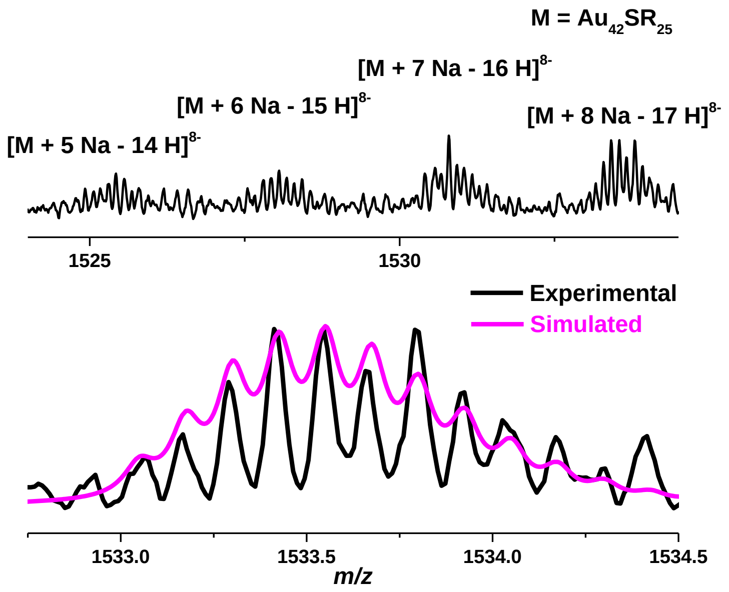

Supplementary Figure 31. Electrospray ionization mass spectrum (top) and isotope patterns (bottom) of $[Au_{42}(SR)_{25} + 8\ Na - 17\ H]^{8-}$ (species **32**) obtained from 12 h sample.

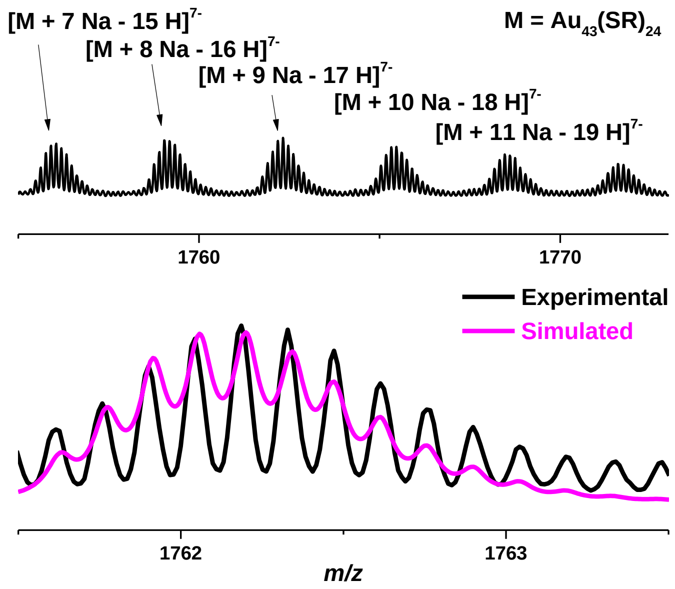

Supplementary Figure 32. Electrospray ionization mass spectrum (top) and isotope patterns (bottom) of $[Au_{43}(SR)_{24} + 9\ Na - 17\ H]^{7-}$ (species **33**) obtained from 12 h sample.

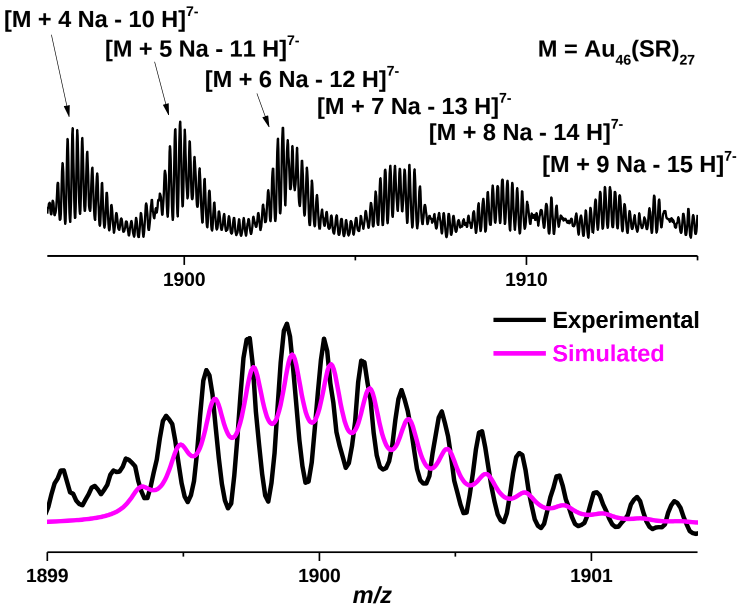

Supplementary Figure 33. Electrospray ionization mass spectrum (top) and isotope patterns (bottom) of $[Au_{46}(SR)_{27} + 5\ Na - 11\ H]^{7-}$ (species **35**) obtained from 12 h sample.

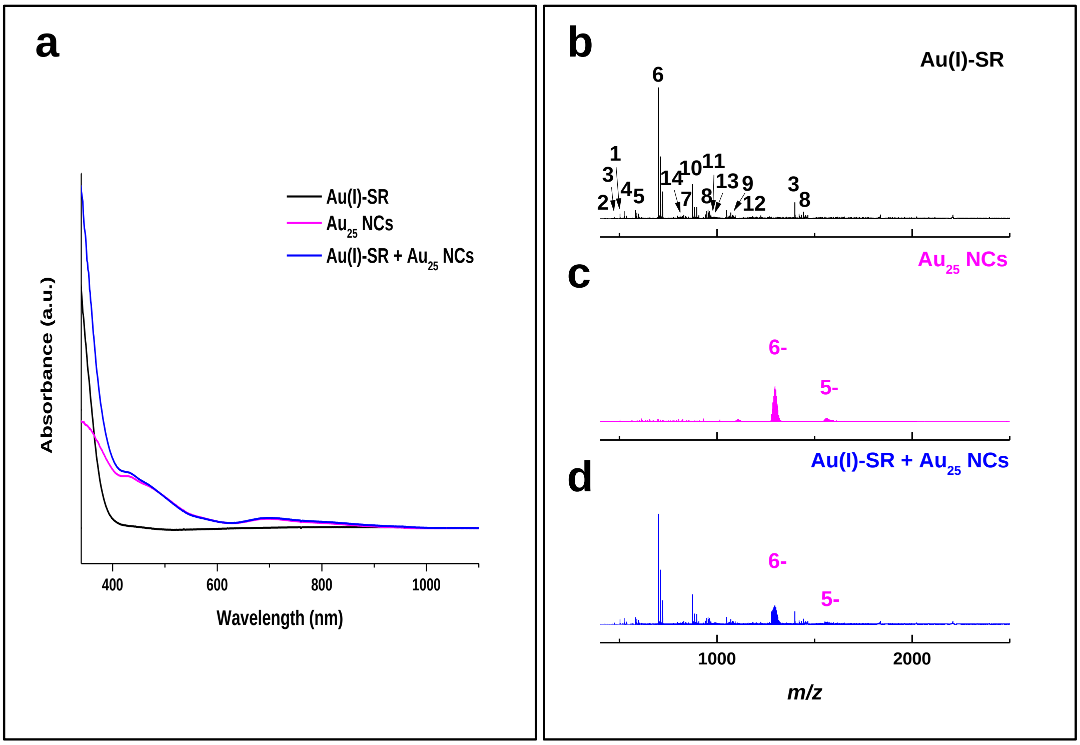

Supplementary Figure 34. (**a**) Ultraviolet-visible absorption and (**b-d**) electrospray ionization mass spectra of (**b**) Au(I)-SR complexes, (**c**) $[Au_{25}(SR)_{18}]^-$ and (**d**) their mixture. The Au(I)-SR complex species identified in the mass spectra are listed in Supplementary Table 1.

Supplementary Table 1. Au(I)-SR complex species identified in Supplementary Figure 34b

Au(I)-SR Complex Species							
**1**	$[Au(SR)_2]^-$	**5**	$[Au_5(SR)_5]^0$	**9**	$[Au_9(SR)_9]^0$	**13**	$[Au_{14}(SR)_{14}]^0$
**2**	$[Au_2(SR)_3]^-$	**6**	$[Au_6(SR)_6]^0$	**10**	$[Au_{10}(SR)_{10}]^0$		
**3**	$[Au_4(SR)_4]^0$	**7**	$[Au_7(SR)_7]^0$	**11**	$[Au_{11}(SR)_{11}]^0$		
**4**	$[Au_4(SR)_5]^-$	**8**	$[Au_8(SR)_8]^0$	**12**	$[Au_{13}(SR)_{13}]^0$		

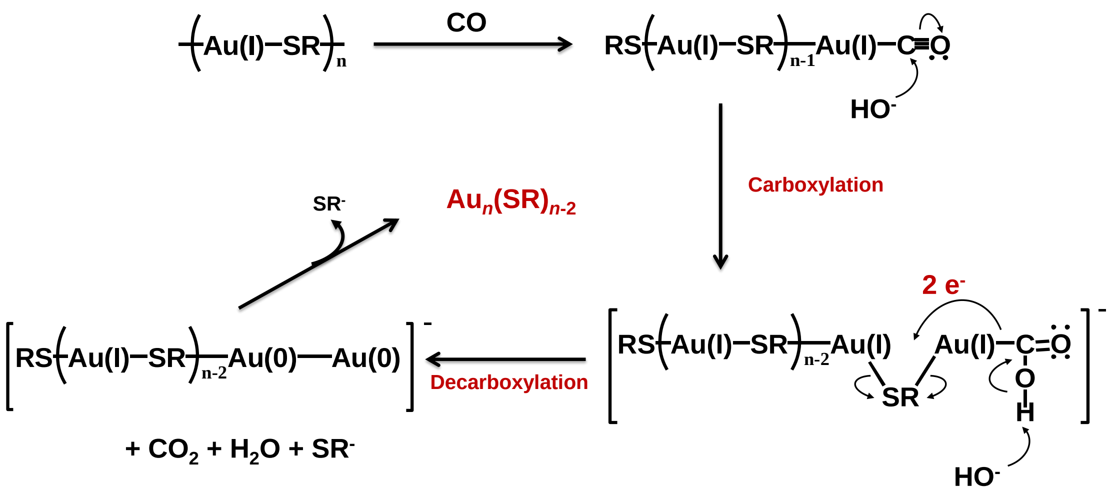

Supplementary Figure 35. Schematic illustration of carboxylation-decarboxylation assisted reduction of Au(I)-SR complexes (exemplified by $[Au_n(SR)_n]^0$ species).

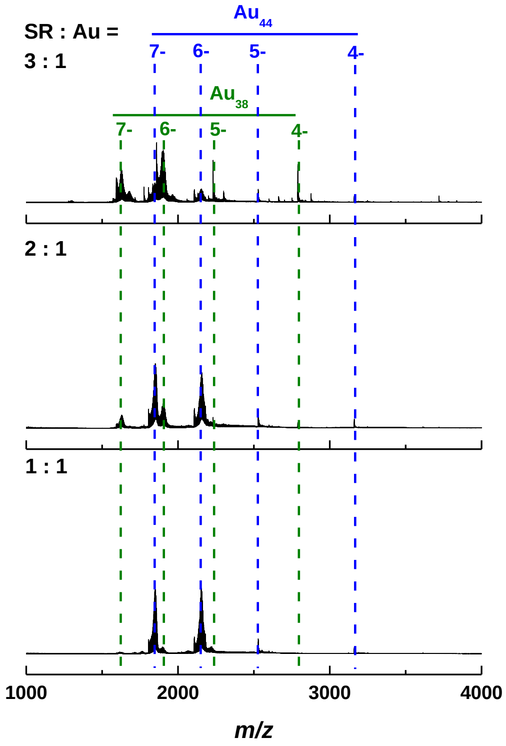

Supplementary Figure 36. Electrospray ionization mass spectra of NC species synthesized with Au(I)-SR complexes formed at varied SR-to-Au ratios. Reaction pH is 13.0. The dotted lines are visual guides for cluster ions of $[Au_{38}(SR)_{24}]^{0}$ (olive lines) and $[Au_{44}(SR)_{26}]^{2-}$ (blue lines) carrying varied charges.

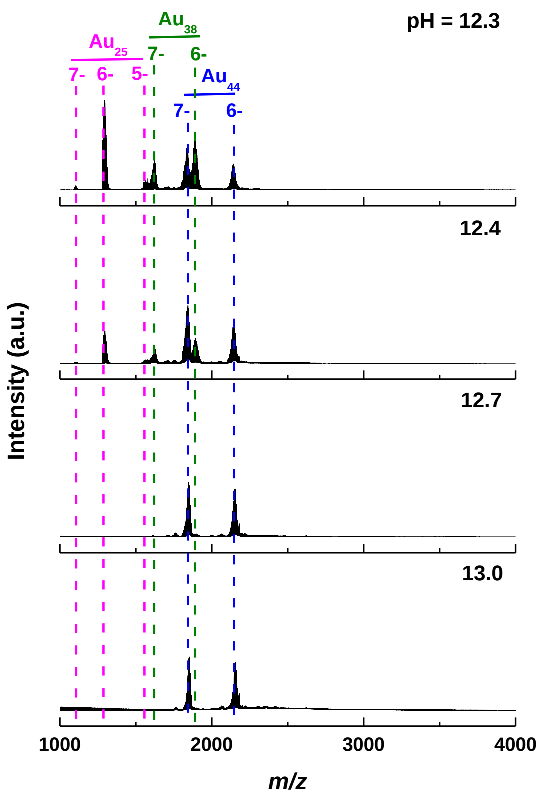

Supplementary Figure 37. Electrospray ionization mass spectra of NC species synthesized at varied pH values. The SR-to-Au ratio used in the synthesis is 1:1. The dotted lines are visual guides for cluster ions of $[Au_{25}(SR)_{18}]^{-}$ (magenta lines), $[Au_{38}(SR)_{24}]^{0}$ (olive lines), and $[Au_{44}(SR)_{26}]^{2-}$ (blue lines) carrying varied charges.

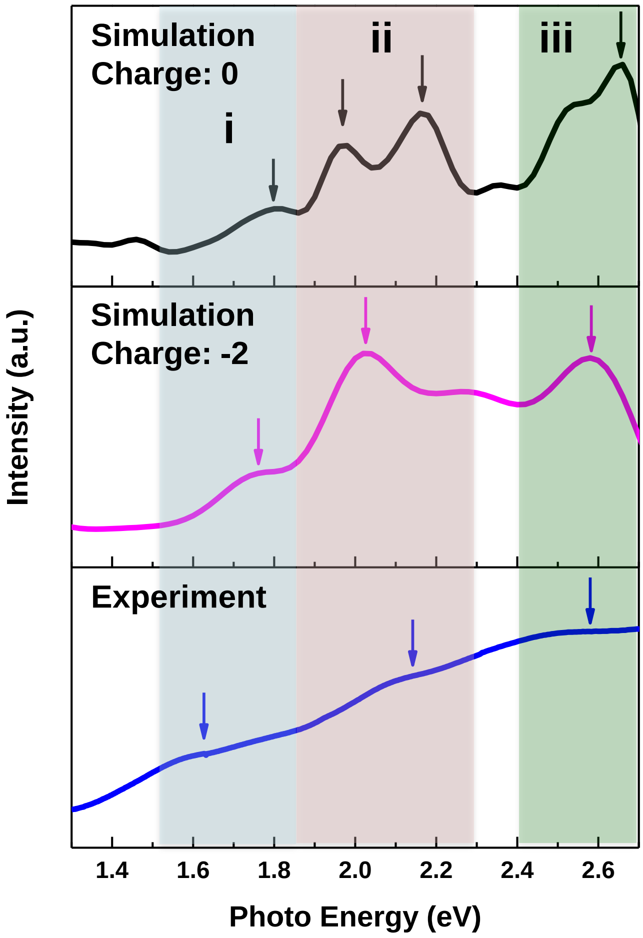

Supplementary Figure 38. Simulated optical absorption spectra of $[Au_{44}(SR)_{26}]^q$ for q = 0 (neutral) and -2 (dianion), in comparison with the experimental spectrum of $[Au_{44}(SR)_{26}]^{2-}$. Simulation was done at the TDDFT-B3LYP/def2-SV(P) level for $[Au_{44}(SCH_3)_{26}]^q$. The main absorption peaks are highlighted in regions i, ii and iii, respectively, for easy comparison.

Supplementary Notes for Stage 0

The formation of $[Au_{25}(SR)_{18}]^-$ was based on a reduction-growth mechanism detailed in our previous publication[1]. The reaction was initiated by CO-reduction of reactive Au(I)-SR complex/NC species via a carboxylation-decarboxylation process. We first exemplified the reduction-growth mechanism by the formation of $[Au_{15}(SR)_{13}]^0$ (N^* = 2) by CO-reduction of $[Au_{10}(SR)_{10}]^0$ (N^* = 0). As shown in Supplementary Note 1, $[Au_{10}(SR)_{10}]^0$ would first associate with a CO to form $[Au_{10}(SR)_{10}CO]^0$ adduct, which could be converted to $[Au_{10}(SR)_{10}COOH]^-$ via a typical Hieber base reaction at alkaline condition (pH = 13.0). Decarboxylation of such adduct then transfers 2 e^- to neighbored Au(I) centers and simultaneously releases two free SR ligands, giving rise to $[Au_{10}(SR)_8]^0$ which contains two Au(0) centers. A plausible reaction pathway for such carboxylation-decarboxylation process can be seen in Supplementary Fig. 35. The as-formed $[Au_{10}(SR)_8]^0$ (N^* = 2) could then associate with a $[Au_5(SR)_5]^0$ to form a stable 2 e^- species $[Au_{15}(SR)_{13}]^0$. The as-formed 2 e^- NC species (e.g., $[Au_{15}(SR)_{13}]^0$) could further grow into NC species with higher N^* values (e.g., N^* = 4 → 6 → 8 → 10) via a similar carboxylation-decarboxylation mechanism. For example, $[Au_{15}(SR)_{13}]^0$ (N^* = 2) could form $[Au_{20}(SR)_{16}]^0$ (N^* = 4) by reacting with a CO and a $[Au_5(SR)_5]^0$. $[Au_{20}(SR)_{16}]^0$ could then be further reduced by one equiv. CO to give rise to a stable 6 e^- NC, $[Au_{20}(SR)_{14}]^0$ (N^* = 6).

Supplementary Note 1. Proposed formation reaction pathways of $[Au_{15}(SR)_{13}]^0$ by CO-reduction of $[Au_{10}(SR)_{10}]^0$.

Association of CO: $[Au_{10}(SR)_{10}]^0 + CO \rightarrow [Au_{10}(SR)_{10}CO]^0$

Hieber Base Reaction: $[Au_{10}(SR)_{10}CO]^0 + OH^- \rightarrow [Au_{10}(SR)_{10}COOH]^-$

Decarboxylation: $[Au_{10}(SR)_{10}COOH]^- + 3\ OH^- \rightarrow [Au_{10}(SR)_8]^0 + CO_3^{2-} + 2\ H_2O + 2\ SR^-$

Size Growth: $[Au_{10}(SR)_8]^0 + [Au_5(SR)_5]^0 \rightarrow [Au_{15}(SR)_{13}]^0$

Overall: $[Au_{10}(SR)_{10}]^0$ ($N^* = 0$) + $[Au_5(SR)_5]^0$ ($N^* = 0$) + CO + 4 OH^- $\rightarrow$ $[Au_{15}(SR)_{13}]^0$ ($N^* = 2$) + CO_3^{2-} + 2 H_2O + 2 SR^-

Given that CO-reduction of Au(I)-SR complexes resulted in a mixture of $[Au_n(SR)_m]^q$ NCs with varied N^* values ($N^* = 2$–10), a size-focusing process of these mix-sized $[Au_n(SR)_m]^q$ could lead to the formation of $[Au_{25}(SR)_{18}]^-$. Besides the abovementioned reduction-growth reaction, the size-focusing process may also involve a couple of other reactions, such as isoelectronic addition, isoelectronic etching, and comproportionation. Isoelectronic addition refers to a reaction where the size of NC increases by associating 0 e^- Au(I)-SR complexes while the valence electron count keeps unchanged. For instance, 8 e^- $[Au_{21}(SR)_{13}]^0$ could associate with a $[Au_4(SR)_5]^-$, giving rise to $[Au_{25}(SR)_{18}]^-$ (Isoelectronic Addition, Supplementary Note 2). By the contrary, in a typical isoelectronic etching reaction (Isoelectronic Etching, Supplementary Note 2), free thiolate ligands would attack and split a large-sized NC, resulting in a small-sized NC with the same valence electron count and a 0 e- Au(I)-SR complex. Besides the isoelectronic reactions, a typical

comproportionation reaction is also shown in Supplementary Note 2, where a NC with moderate size and N^* value (e.g., $[Au_{25}(SR)_{18}]^-$) is produced at the expense of a NC with larger size and N^* value (e.g., $[Au_{28}(SR)_{21}]^{3-}$) together with a NC with smaller size and N^* value (e.g., $[Au_{22}(SR)_{17}]^-$).

Supplementary Note 2. Proposed isoelectronic addition, isoelectronic etching, and comproportionation reactions occurring in the formation of $[Au_{25}(SR)_{18}]^-$.

Isoelectronic Addition: $[Au_{21}(SR)_{13}]^0$ (N^* = 8) + $[Au_4(SR)_5]^-$ (N^* = 0) → $[Au_{25}(SR)_{18}]^-$ (N^* = 8)

Isoelectronic Etching: $[Au_{29}(SR)_{21}]^0$ (N^* = 8) + SR^- → $[Au_{25}(SR)_{18}]^-$ (N^* = 8) + $[Au_4(SR)_4]^0$ (N^* = 0)

Comproportionation: $[Au_{28}(SR)_{21}]^{3-}$ (N^* = 10) + $[Au_{22}(SR)_{17}]^-$ (N^* = 6) → 2 $[Au_{25}(SR)_{18}]^-$ (N^* = 8) + 2 SR^-

Supplementary Notes for Stage I

Supplementary Note 3. Proposed mechanism for Au_{25}-mediated formation of $[Au_{28}(SR)_{21}]^{3-}$ via LaMer-like mechanism.

Association of CO: $[Au_{25}(SR)_{18}]^{-} + CO \rightarrow [Au_{25}(SR)_{18}CO]^{-}$

Reduction-Growth: $[Au_{25}(SR)_{18}CO]^{-} + [Au_4(SR)_5]^{-} + 4\ OH^{-} \rightarrow [Au_{29}(SR)_{23}]^{4-} + CO_3^{2-} + 2\ H_2O$

Splitting: $[Au_{29}(SR)_{23}]^{4-} \rightarrow [Au_{28}(SR)_{21}]^{3-} + [Au(SR)_2]^{-}$

Overall: $[Au_{25}(SR)_{18}]^{-}$ ($N^* = 8$) + $[Au_4(SR)_5]^{-}$ ($N^* = 0$) + CO + 4 OH^{-} $\rightarrow$ $[Au_{28}(SR)_{21}]^{3-}$ ($N^* = 10$) + $[Au(SR)_2]^{-}$ ($N^* = 0$) + CO_3^{2-} + 2 H_2O

Supplementary Note 4. Proposed mechanism for Au_{25}-mediated formation of $[Au_{53}(SR)_{41}]^0$ via aggregative growth mechanism.

Association of CO: $[Au_{25}(SR)_{18}]^- + CO \rightarrow [Au_{25}(SR)_{18}CO]^-$

Reduction-Growth: $[Au_{25}(SR)_{18}CO]^- + [Au_{10}(SR)_{10}]^0 + 4\ OH^- \rightarrow [Au_{35}(SR)_{28}]^{3-} + CO_3^{2-} + 2\ H_2O$

Agglomeration: $[Au_{35}(SR)_{28}]^{3-} + [Au_{18}(SR)_{14}]^{2+} \rightarrow [Au_{53}(SR)_{41}]^0 + SR^-$

Overall: $[Au_{25}(SR)_{18}]^-$ ($N^* = 8$) + $[Au_{18}(SR)_{14}]^{2+}$ ($N^* = 2$) + $[Au_{10}(SR)_{10}]^0$ ($N^* = 0$) + $CO + 4\ OH^- \rightarrow [Au_{53}(SR)_{41}]^0$ ($N^* = 12$) + $CO_3^{2-} + 2\ H_2O + SR^-$

Supplementary Note 5. Proposed mechanism for size evolution from 12 e^- $[Au_{53}(SR)_{41}]^0$ to 20 e^- $[Au_{44}(SR)_{26}]^{2-}$ or $[Au_{46}(SR)_{27}]^-$.

Pathway 1:

$[Au_{53}(SR)_{41}]^0$ (N^* = 12) + CO + 4 OH^- → $[Au_{51}(SR)_{38}]^-$ (N^* = 14) + $[Au_2(SR)_3]^-$ (N^* = 0) + CO_3^{2-} + 2 H_2O

$[Au_{51}(SR)_{38}]^-$ (N^* = 14) + CO + 4 OH^- → $[Au_{42}(SR)_{25}]^+$ (N^* = 16) + $[Au_9(SR)_9]^0$ (N^* = 0) + CO_3^{2-} + 2 H_2O + 4 SR^-

$[Au_{42}(SR)_{25}]^+$ (N^* = 16) + $[Au(SR)_2]^-$ (N^* = 0) + CO + 4 OH^- → $[Au_{43}(SR)_{24}]^+$ (N^* = 18) + CO_3^{2-} + 2 H_2O + 3 SR^-

$[Au_{43}(SR)_{24}]^+$ (N^* = 18) + $[Au(SR)_2]^-$ (N^* = 0) + CO + 4 OH^- → $[Au_{44}(SR)_{26}]^{2-}$ (N^* = 20) + CO_3^{2-} + 2 H_2O

$[Au_{43}(SR)_{24}]^+$ (N^* = 18) + $[Au_4(SR)_5]^-$ (N^* = 0) + CO + 4 OH^- → $[Au_{46}(SR)_{27}]^{2-}$ (N^* = 20) + $[Au(SR)_2]^-$ (N^* = 0) + CO_3^{2-} + 2 H_2O

Pathway 2:

$[Au_{53}(SR)_{41}]^0$ (N^* = 12) + $[Au(SR)_2]^-$ (N^* = 0) + $[Au_4(SR)_5]^-$ (N^* = 0) + 2 CO + 8 OH^- → $[Au_{40}(SR)_{25}]^+$ (N^* = 14) + $[Au_{18}(SR)_{14}]^{2+}$ (N^* = 2) + 2 CO_3^{2-} + 4 H_2O + 9 SR^-

$[Au_{40}(SR)_{25}]^+$ (N^* = 14) + $[Au_2(SR)_3]^-$ (N^* = 0) + CO + 4 OH^- → $[Au_{42}(SR)_{25}]^+$ (N^* = 16) + CO_3^{2-} + 2 H_2O + 3 SR^-

$[Au_{42}(SR)_{25}]^+$ (N^* = 16) + $[Au(SR)_2]^-$ (N^* = 0) + CO + 4 OH^- → $[Au_{43}(SR)_{24}]^+$ (N^* = 18) + CO_3^{2-} + 2 H_2O + 3 SR^-

$[Au_{43}(SR)_{24}]^+$ (N^* = 18) + $[Au(SR)_2]^-$ (N^* = 0) + CO + 4 OH^- → $[Au_{44}(SR)_{26}]^{2-}$ (N^* = 20) + CO_3^{2-} + 2 H_2O

$[Au_{43}(SR)_{24}]^+$ (N^* = 18) + $[Au_4(SR)_5]^-$ (N^* = 0) + CO + 4 OH^- → $[Au_{46}(SR)_{27}]^{2-}$ (N^* = 20) + $[Au(SR)_2]^-$ (N^* = 0) + CO_3^{2-} + 2 H_2O

Supplementary Notes for Stage II

Supplementary Note 6. Proposed model reactions in the thermodynamically controlled size-focusing.

Reduction-Growth: $[Au_{43}(SR)_{24}]^{+}$ (N^* = 18) + $[Au(SR)_2]^{-}$ (N^* = 0) + CO + 4 OH^{-} → $[Au_{44}(SR)_{26}]^{2-}$ (N^* = 20) + CO_3^{2-} + 2 H_2O

Isoelectronic Etching: $[Au_{46}(SR)_{27}]^{-}$ (N^* = 20) + 2 SR^{-} → $[Au_{44}(SR)_{26}]^{2-}$ (N^* = 20) + $[Au_2(SR)_3]^{-}$ (N^* = 0)

Supplementary Notes for Relative Stability of $[Au_{25}(SR)_{18}]^-$ and $[Au_{44}(SR)_{26}]^{2-}$

Supplementary Note 7. Proposed reaction to connect $[Au_{25}(SR)_{18}]^-$ and $[Au_{44}(SR)_{26}]^{2-}$ thermodynamically.

$2\ [Au_{25}(SR)_{18}]^-\ (N^* = 8) + 2\ CO + 8\ OH^- \rightarrow [Au_{44}(SR)_{26}]^{2-}\ (N^* = 20) + 2\ [Au_2(SR)_3]^-\ (N^* = 0) + 2\ [Au(SR)_2]^-\ (N^* = 0) + 2\ CO_3^{2-} + 4\ H_2O$

$\boldsymbol{\Delta H}$ = -400 kcal per mol of $[Au_{44}(SR)_{26}]^{2-}$ or -200 kcal per mol of $[Au_{25}(SR)_{18}]^-$

To compute this energy, the energy of each species in a Conductor-like Screening Model (COSMO) solvation model was obtained at the DFT-TPSS/def2-SV(P) level[2-5].

Supplementary Discussion

Proposed formation and consumption pathways for each NC species are detailed in this section.

2 e^-, $[Au_{10}(SR)_8]^0$

Formation:

- $2\ [Au_5(SR)_5]^0 + CO + 4\ OH^- \rightarrow [Au_{10}(SR)_8]^0 + CO_3^{2-} + 2\ H_2O + 2\ SR^-$
- $[Au_6(SR)_6]^0 + [Au_4(SR)_4]^0 + CO + 4\ OH^- \rightarrow [Au_{10}(SR)_8]^0 + CO_3^{2-} + 2\ H_2O + 2\ SR^-$
- $[Au_6(SR)_6]^0 + [Au_4(SR)_5]^- + CO + 4\ OH^- \rightarrow [Au_{10}(SR)_8]^0 + CO_3^{2-} + 2\ H_2O + 3\ SR^-$
- $[Au_8(SR)_8]^0 + [Au_2(SR)_3]^- + CO + 4\ OH^- \rightarrow [Au_{10}(SR)_8]^0 + CO_3^{2-} + 2\ H_2O + 3\ SR^-$
- $[Au_9(SR)_9]^0 + [Au(SR)_2]^- + CO + 4\ OH^- \rightarrow [Au_{10}(SR)_8]^0 + CO_3^{2-} + 2\ H_2O + 3\ SR^-$
- $[Au_{10}(SR)_{10}]^0 + CO + 4\ OH^- \rightarrow [Au_{10}(SR)_8]^0 + CO_3^{2-} + 2\ H_2O + 2\ SR^-$

Consumption:

- $[Au_{10}(SR)_8]^0 + [Au_5(SR)_5]^0 \rightarrow [Au_{15}(SR)_{13}]^0$
- $[Au_{10}(SR)_8]^0 + [Au_8(SR)_8]^0 \rightarrow [Au_{18}(SR)_{14}]^{2+} + 2\ SR^-$
- $2\ [Au_{10}(SR)_8]^0 + CO + 4\ OH^- \rightarrow [Au_{20}(SR)_{14}]^0 + CO_3^{2-} + 2\ H_2O + 2\ SR^-$

2 e^-, $[Au_{15}(SR)_{13}]^0$

Formation:

- $[Au_8(SR)_8]^0 + [Au_7(SR)_7]^0 + CO + 4\ OH^- \rightarrow [Au_{15}(SR)_{13}]^0 + CO_3^{2-} + 2\ H_2O + 2\ SR^-$
- $[Au_9(SR)_9]^0 + [Au_6(SR)_6]^0 + CO + 4\ OH^- \rightarrow [Au_{15}(SR)_{13}]^0 + CO_3^{2-} + 2\ H_2O + 2\ SR^-$
- $[Au_{11}(SR)_{11}]^0 + [Au_4(SR)_4]^0 + CO + 4\ OH^- \rightarrow [Au_{15}(SR)_{13}]^0 + CO_3^{2-} + 2\ H_2O + 2\ SR^-$
- $[Au_{11}(SR)_{11}]^0 + [Au_4(SR)_5]^- + CO + 4\ OH^- \rightarrow [Au_{15}(SR)_{13}]^0 + CO_3^{2-} + 2\ H_2O + 3\ SR^-$
- $[Au_{13}(SR)_{13}]^0 + [Au_2(SR)_3]^- + CO + 4\ OH^- \rightarrow [Au_{15}(SR)_{13}]^0 + CO_3^{2-} + 2\ H_2O + 3\ SR^-$
- $[Au_{14}(SR)_{14}]^0 + [Au(SR)_2]^- + CO + 4\ OH^- \rightarrow [Au_{15}(SR)_{13}]^0 + CO_3^{2-} + 2\ H_2O + 3\ SR^-$
- $[Au_{10}(SR)_8]^0 + [Au_5(SR)_5]^0 \rightarrow [Au_{15}(SR)_{13}]^0$

Consumption:

- $[Au_{15}(SR)_{13}]^0 + [Au_5(SR)_5]^0 + 2\ CO + 8\ OH^- \rightarrow [Au_{20}(SR)_{14}]^0 + 2\ CO_3^{2-} + 4\ H_2O + 4\ SR^-$
- $[Au_{15}(SR)_{13}]^0 + [Au_7(SR)_7]^0 + 2\ CO + 8\ OH^- \rightarrow [Au_{22}(SR)_{17}]^- + 2\ CO_3^{2-} + 4\ H_2O + 3\ SR^-$
- $[Au_{15}(SR)_{13}]^0 + [Au_{10}(SR)_8]^0 + 2\ CO + 8\ OH^- \rightarrow [Au_{25}(SR)_{17}]^0 + 2\ CO_3^{2-} + 4\ H_2O + 4\ SR^-$
- $[Au_{15}(SR)_{13}]^0 + [Au_{10}(SR)_8]^0 + 2\ CO + 8\ OH^- \rightarrow [Au_{25}(SR)_{18}]^- + 2\ CO_3^{2-} + 4\ H_2O + 3\ SR^-$

2 e$^-$, $[Au_{18}(SR)_{14}]^{2+}$

Formation:

- $2\ [Au_9(SR)_9]^0 + CO + 4\ OH^- \rightarrow [Au_{18}(SR)_{14}]^{2+} + CO_3^{2-} + 2\ H_2O + 4\ SR^-$
- $[Au_{11}(SR)_{11}]^0 + [Au_7(SR)_7]^0 + CO + 4\ OH^- \rightarrow [Au_{18}(SR)_{14}]^{2+} + CO_3^{2-} + 2\ H_2O + 4\ SR^-$
- $[Au_{13}(SR)_{13}]^0 + [Au_5(SR)_5]^0 + CO + 4\ OH^- \rightarrow [Au_{18}(SR)_{14}]^{2+} + CO_3^{2-} + 2\ H_2O + 4\ SR^-$
- $[Au_{14}(SR)_{14}]^0 + [Au_4(SR)_4]^0 + CO + 4\ OH^- \rightarrow [Au_{18}(SR)_{14}]^{2+} + CO_3^{2-} + 2\ H_2O + 4\ SR^-$
- $[Au_{14}(SR)_{14}]^0 + [Au_4(SR)_5]^- + CO + 4\ OH^- \rightarrow [Au_{18}(SR)_{14}]^{2+} + CO_3^{2-} + 2\ H_2O + 5\ SR^-$
- $[Au_{10}(SR)_8]^0 + [Au_8(SR)_8]^0 \rightarrow [Au_{18}(SR)_{14}]^{2+} + 2\ SR^-$
- $[Au_{53}(SR)_{41}]^0 + [Au(SR)_2]^- + [Au_4(SR)_4]^0 + 2\ CO + 8\ OH^- \rightarrow [Au_{40}(SR)_{25}]^+ + [Au_{18}(SR)_{14}]^{2+} + 2\ CO_3^{2-} + 4\ H_2O + 8\ SR^-$
- $[Au_{53}(SR)_{41}]^0 + [Au(SR)_2]^- + [Au_4(SR)_5]^- + 2\ CO + 8\ OH^- \rightarrow [Au_{40}(SR)_{25}]^+ + [Au_{18}(SR)_{14}]^{2+} + 2\ CO_3^{2-} + 4\ H_2O + 9\ SR^-$

Consumption:

- $[Au_{18}(SR)_{14}]^{2+} + [Au_2(SR)_3]^- + 2\ CO + 8\ OH^- \rightarrow [Au_{20}(SR)_{14}]^0 + 2\ CO_3^{2-} + 4\ H_2O + 3\ SR^-$
- $[Au_{18}(SR)_{14}]^{2+} + [Au_4(SR)_4]^0 + 2\ CO + 8\ OH^- \rightarrow [Au_{22}(SR)_{17}]^- + 2\ CO_3^{2-} + 4\ H_2O + SR^-$
- $[Au_{18}(SR)_{14}]^{2+} + [Au_4(SR)_5]^- + 2\ CO + 8\ OH^- \rightarrow [Au_{22}(SR)_{17}]^- + 2\ CO_3^{2-} + 4\ H_2O + 2\ SR^-$
- $[Au_{18}(SR)_{14}]^{2+} + [Au_{25}(SR)_{18}]^- + [Au_{10}(SR)_{10}]^0 + CO + 4\ OH^- \rightarrow [Au_{53}(SR)_{41}]^0 + CO_3^{2-} + 2\ H_2O + SR^-$

6 e$^-$, $[Au_{20}(SR)_{14}]^0$

Formation:

- 2 $[Au_{10}(SR)_8]^0$ + CO + 4 OH^- → $[Au_{20}(SR)_{14}]^0$ + CO_3^{2-} + 2 H_2O + 2 SR^-
- $[Au_{15}(SR)_{13}]^0$ + $[Au_5(SR)_5]^0$ + 2 CO + 8 OH^- → $[Au_{20}(SR)_{14}]^0$ + 2 CO_3^{2-} + 4 H_2O + 4 SR^-
- $[Au_{18}(SR)_{14}]^{2+}$ + $[Au_2(SR)_3]^-$ + 2 CO + 8 OH^- → $[Au_{20}(SR)_{14}]^0$ + 2 CO_3^{2-} + 4 H_2O + 3 SR^-

Consumption:

- $[Au_{20}(SR)_{14}]^0$ + $[Au_2(SR)_3]^-$ → $[Au_{22}(SR)_{17}]^-$
- $[Au_{20}(SR)_{14}]^0$ + $[Au(SR)_2]^-$ + CO + 4 OH^- → $[Au_{21}(SR)_{13}]^0$ + CO_3^{2-} + 2 H_2O + 3 SR^-
- $[Au_{20}(SR)_{14}]^0$ + $[Au_4(SR)_4]^0$ + CO + 4 OH^- → $[Au_{24}(SR)_{16}]^0$ + CO_3^{2-} + 2 H_2O + 2 SR^-
- $[Au_{20}(SR)_{14}]^0$ + $[Au_4(SR)_5]^-$ + CO + 4 OH^- → $[Au_{24}(SR)_{16}]^0$ + CO_3^{2-} + 2 H_2O + 3 SR^-
- $[Au_{20}(SR)_{14}]^0$ + $[Au_5(SR)_5]^0$ + CO + 4 OH^- → $[Au_{25}(SR)_{17}]^0$ + CO_3^{2-} + 2 H_2O + 2 SR^-
- $[Au_{20}(SR)_{14}]^0$ + $[Au_5(SR)_5]^0$ + CO + 4 OH^- → $[Au_{25}(SR)_{18}]^-$ + CO_3^{2-} + 2 H_2O + SR^-
- $[Au_{20}(SR)_{14}]^0$ + $[Au_9(SR)_9]^0$ + CO + 4 OH^- → $[Au_{29}(SR)_{21}]^0$ + CO_3^{2-} + 2 H_2O + 2 SR^-
- $[Au_{33}(SR)_{22}]^-$ + $[Au_{20}(SR)_{14}]^0$ → $[Au_{43}(SR)_{24}]^+$ + $[Au_{10}(SR)_{10}]^0$ + 2 SR^-

6 e$^-$, $[Au_{22}(SR)_{17}]^-$

Formation:

- $[Au_{15}(SR)_{13}]^0 + [Au_7(SR)_7]^0 + 2\ CO + 8\ OH^- \rightarrow [Au_{22}(SR)_{17}]^- + 2\ CO_3^{2-} + 4\ H_2O + 3\ SR^-$
- $[Au_{18}(SR)_{14}]^{2+} + [Au_4(SR)_4]^0 + 2\ CO + 8\ OH^- \rightarrow [Au_{22}(SR)_{17}]^- + 2\ CO_3^{2-} + 4\ H_2O + SR^-$
- $[Au_{18}(SR)_{14}]^{2+} + [Au_4(SR)_5]^- + 2\ CO + 8\ OH^- \rightarrow [Au_{22}(SR)_{17}]^- + 2\ CO_3^{2-} + 4\ H_2O + 2\ SR^-$
- $[Au_{20}(SR)_{14}]^0 + [Au_2(SR)_3]^- \rightarrow [Au_{22}(SR)_{17}]^-$

Consumption:

- $[Au_{22}(SR)_{17}]^- + [Au_2(SR)_3]^- + CO + 4\ OH^- \rightarrow [Au_{24}(SR)_{16}]^0 + CO_3^{2-} + 2\ H_2O + 4\ SR^-$
- $[Au_{22}(SR)_{17}]^- + [Au_7(SR)_7]^0 + CO + 4\ OH^- \rightarrow [Au_{29}(SR)_{21}]^0 + CO_3^{2-} + 2\ H_2O + 3\ SR^-$
- $[Au_{28}(SR)_{21}]^{3-} + [Au_{22}(SR)_{17}]^- \rightarrow 2\ [Au_{25}(SR)_{18}]^- + 2\ SR^-$

8 e$^-$, $[Au_{21}(SR)_{13}]^0$

Formation:

- $[Au_{20}(SR)_{14}]^0 + [Au(SR)_2]^- + CO + 4\ OH^- \rightarrow [Au_{21}(SR)_{13}]^0 + CO_3^{2-} + 2\ H_2O + 3\ SR^-$

Consumption:

- $[Au_{21}(SR)_{13}]^0 + [Au_4(SR)_4]^0 \rightarrow [Au_{25}(SR)_{17}]^0$
- $[Au_{21}(SR)_{13}]^0 + [Au_4(SR)_5]^- \rightarrow [Au_{25}(SR)_{18}]^-$
- $[Au_{21}(SR)_{13}]^0 + [Au_8(SR)_8]^0 \rightarrow [Au_{29}(SR)_{21}]^0$

8 e^-, $[Au_{24}(SR)_{16}]^0$

Formation:

- $[Au_{20}(SR)_{14}]^0 + [Au_4(SR)_4]^0 + CO + 4\ OH^- \rightarrow [Au_{24}(SR)_{16}]^0 + CO_3^{2-} + 2\ H_2O + 2\ SR^-$
- $[Au_{20}(SR)_{14}]^0 + [Au_4(SR)_5]^- + CO + 4\ OH^- \rightarrow [Au_{24}(SR)_{16}]^0 + CO_3^{2-} + 2\ H_2O + 3\ SR^-$
- $[Au_{22}(SR)_{17}]^- + [Au_2(SR)_3]^- + CO + 4\ OH^- \rightarrow [Au_{24}(SR)_{16}]^0 + CO_3^{2-} + 2\ H_2O + 4\ SR^-$

Consumption:

- $[Au_{24}(SR)_{16}]^0 + [Au(SR)_2]^- \rightarrow [Au_{25}(SR)_{18}]^-$
- $[Au_{24}(SR)_{16}]^0 + [Au_5(SR)_5]^0 \rightarrow [Au_{29}(SR)_{21}]^0$
- $[Au_{24}(SR)_{16}]^0 + CO + 4\ OH^- \rightarrow [Au_{24}(SR)_{14}]^0 + CO_3^{2-} + 2\ H_2O + 2\ SR^-$

8 e^-, $[Au_{25}(SR)_{17}]^0$

Formation:

- $[Au_{15}(SR)_{13}]^0 + [Au_{10}(SR)_8]^0 + 2\ CO + 8\ OH^- \rightarrow [Au_{25}(SR)_{17}]^0 + 2\ CO_3^{2-} + 4\ H_2O + 4\ SR^-$
- $[Au_{20}(SR)_{14}]^0 + [Au_5(SR)_5]^0 + CO + 4\ OH^- \rightarrow [Au_{25}(SR)_{17}]^0 + CO_3^{2-} + 2\ H_2O + 2\ SR^-$
- $[Au_{21}(SR)_{13}]^0 + [Au_4(SR)_4]^0 \rightarrow [Au_{25}(SR)_{17}]^0$
- $[Au_{25}(SR)_{18}]^- + [Au_4(SR)_4]^0 \rightarrow [Au_{25}(SR)_{17}]^0 + [Au_4(SR)_5]^-$

Consumption:

- $[Au_{25}(SR)_{17}]^0 + SR^- \rightarrow [Au_{25}(SR)_{18}]^-$
- $[Au_{25}(SR)_{17}]^0 + [Au_4(SR)_4]^0 \rightarrow [Au_{29}(SR)_{21}]^0$

8 e^-, $[Au_{25}(SR)_{18}]^-$

Formation:

- $[Au_{15}(SR)_{13}]^0 + [Au_{10}(SR)_8]^0 + 2\ CO + 8\ OH^- \rightarrow [Au_{25}(SR)_{18}]^- + 2\ CO_3^{2-} + 4\ H_2O + 3\ SR^-$
- $[Au_{20}(SR)_{14}]^0 + [Au_5(SR)_5]^0 + CO + 4\ OH^- \rightarrow [Au_{25}(SR)_{18}]^- + CO_3^{2-} + 2\ H_2O + SR^-$
- $[Au_{21}(SR)_{13}]^0 + [Au_4(SR)_5]^- \rightarrow [Au_{25}(SR)_{18}]^-$
- $[Au_{24}(SR)_{16}]^0 + [Au(SR)_2]^- \rightarrow [Au_{25}(SR)_{18}]^-$
- $[Au_{25}(SR)_{17}]^0 + SR^- \rightarrow [Au_{25}(SR)_{18}]^-$
- $[Au_{29}(SR)_{21}]^0 + SR^- \rightarrow [Au_{25}(SR)_{18}]^- + [Au_4(SR)_4]^0$
- $[Au_{28}(SR)_{21}]^{3-} + [Au_{22}(SR)_{17}]^- \rightarrow 2\ [Au_{25}(SR)_{18}]^- + 2\ SR^-$

Consumption:

- $[Au_{25}(SR)_{18}]^- + [Au_4(SR)_4]^0 \rightarrow [Au_{25}(SR)_{17}]^0 + [Au_4(SR)_5]^-$
- $[Au_{25}(SR)_{18}]^- + [Au_4(SR)_5]^- + CO + 4\ OH^- \rightarrow [Au_{28}(SR)_{21}]^{3-} + [Au(SR)_2]^- + CO_3^{2-} + 2\ H_2O$
- $[Au_{25}(SR)_{18}]^- + [Au_{18}(SR)_{14}]^{2+} + [Au_{10}(SR)_{10}]^0 + CO + 4\ OH^- \rightarrow [Au_{53}(SR)_{41}]^0 + CO_3^{2-} + 2\ H_2O + SR^-$

8 e^-, $[Au_{29}(SR)_{21}]^0$

Formation:

- $[Au_{20}(SR)_{14}]^0 + [Au_9(SR)_9]^0 + CO + 4\ OH^- \rightarrow [Au_{29}(SR)_{21}]^0 + CO_3^{2-} + 2\ H_2O + 2\ SR^-$
- $[Au_{22}(SR)_{17}]^- + [Au_7(SR)_7]^0 + CO + 4\ OH^- \rightarrow [Au_{29}(SR)_{21}]^0 + CO_3^{2-} + 2\ H_2O + 3\ SR^-$
- $[Au_{21}(SR)_{13}]^0 + [Au_8(SR)_8]^0 \rightarrow [Au_{29}(SR)_{21}]^0$
- $[Au_{24}(SR)_{16}]^0 + [Au_5(SR)_5]^0 \rightarrow [Au_{29}(SR)_{21}]^0$
- $[Au_{25}(SR)_{17}]^0 + [Au_4(SR)_4]^0 \rightarrow [Au_{29}(SR)_{21}]^0$

Consumption:

- $[Au_{29}(SR)_{21}]^- + SR^- \rightarrow [Au_{25}(SR)_{18}]^- + [Au_4(SR)_4]^0$

10 e^-, $[Au_{24}(SR)_{14}]^0$

Formation:

- $[Au_{24}(SR)_{16}]^0 + CO + 4\ OH^- \rightarrow [Au_{24}(SR)_{14}]^0 + CO_3^{2-} + 2\ H_2O + 2\ SR^-$

Consumption:

- $[Au_{24}(SR)_{14}]^0 + [Au_4(SR)_4]^0 + 3\ SR^- \rightarrow [Au_{28}(SR)_{21}]^{3-}$
- $[Au_{24}(SR)_{14}]^0 + [Au_4(SR)_5]^- + 2\ SR^- \rightarrow [Au_{28}(SR)_{21}]^{3-}$

10 e^-, $[Au_{28}(SR)_{21}]^{3-}$

Formation:

- $[Au_{25}(SR)_{18}]^- + [Au_4(SR)_5]^- + CO + 4\ OH^- \rightarrow [Au_{28}(SR)_{21}]^{3-} + [Au(SR)_2]^- + CO_3^{2-} + 2\ H_2O$
- $[Au_{24}(SR)_{14}]^0 + [Au_4(SR)_4]^0 + 3\ SR^- \rightarrow [Au_{28}(SR)_{21}]^{3-}$
- $[Au_{24}(SR)_{14}]^0 + [Au_4(SR)_5]^- + 2\ SR^- \rightarrow [Au_{28}(SR)_{21}]^{3-}$

Consumption:

- $[Au_{28}(SR)_{21}]^{3-} + [Au_5(SR)_5]^0 + CO + 4\ OH^- \rightarrow [Au_{33}(SR)_{22}]^- + CO_3^{2-} + 2\ H_2O + 4\ SR^-$
- $[Au_{28}(SR)_{21}]^{3-} + [Au_{22}(SR)_{17}]^- \rightarrow 2\ [Au_{25}(SR)_{18}]^- + 2\ SR^-$

12 e^-, $[Au_{33}(SR)_{22}]^-$

Formation:

- $[Au_{28}(SR)_{21}]^{3-}$ + $[Au_5(SR)_5]^0$ + CO + 4 OH^- → $[Au_{33}(SR)_{22}]^-$ + CO_3^{2-} + 2 H_2O + 4 SR^-

Consumption:

- $[Au_{33}(SR)_{22}]^-$ + $[Au_4(SR)_4]^0$ + CO + 4 OH^- → $[Au_{37}(SR)_{23}]^0$ + CO_3^{2-} + 2 H_2O + 3 SR^-
- $[Au_{33}(SR)_{22}]^-$ + $[Au_4(SR)_5]^-$ + CO + 4 OH^- → $[Au_{37}(SR)_{23}]^0$ + CO_3^{2-} + 2 H_2O + 4 SR^-
- $[Au_{33}(SR)_{22}]^-$ + $[Au_5(SR)_5]^0$ + CO + 4 OH^- → $[Au_{38}(SR)_{24}]^0$ + CO_3^{2-} + 2 H_2O + 3 SR^-
- $[Au_{33}(SR)_{22}]^-$ + $[Au_{20}(SR)_{14}]^0$ → $[Au_{43}(SR)_{24}]^+$ + $[Au_{10}(SR)_{10}]^0$ + 2 SR^-

12 e^-, $[Au_{53}(SR)_{41}]^0$

Formation:

- $[Au_{25}(SR)_{18}]^- + [Au_{18}(SR)_{14}]^{2+} + [Au_{10}(SR)_{10}]^0 + CO + 4\ OH^- \rightarrow [Au_{53}(SR)_{41}]^0 + CO_3^{2-} + 2\ H_2O + SR^-$

Consumption:

- $[Au_{53}(SR)_{41}]^0 + CO + 4\ OH^- \rightarrow [Au_{51}(SR)_{38}]^- + [Au_2(SR)_3]^- + CO_3^{2-} + 2\ H_2O$
- $[Au_{53}(SR)_{41}]^0 + [Au(SR)_2]^- + [Au_4(SR)_4]^0 + 2\ CO + 8\ OH^- \rightarrow [Au_{40}(SR)_{25}]^+ + [Au_{18}(SR)_{14}]^{2+} + 2\ CO_3^{2-} + 4\ H_2O + 8\ SR^-$
- $[Au_{53}(SR)_{41}]^0 + [Au(SR)_2]^- + [Au_4(SR)_5]^- + 2\ CO + 8\ OH^- \rightarrow [Au_{40}(SR)_{25}]^+ + [Au_{18}(SR)_{14}]^{2+} + 2\ CO_3^{2-} + 4\ H_2O + 9\ SR^-$

14 e^-, $[Au_{37}(SR)_{23}]^0$

Formation:

- $[Au_{33}(SR)_{22}]^- + [Au_4(SR)_4]^0 + CO + 4\ OH^- \rightarrow [Au_{37}(SR)_{23}]^0 + CO_3^{2-} + 2\ H_2O + 3\ SR^-$
- $[Au_{33}(SR)_{22}]^- + [Au_4(SR)_5]^- + CO + 4\ OH^- \rightarrow [Au_{37}(SR)_{23}]^0 + CO_3^{2-} + 2\ H_2O + 4\ SR^-$

Consumption:

- $[Au_{37}(SR)_{23}]^0 + [Au(SR)_2]^- \rightarrow [Au_{38}(SR)_{24}]^0 + SR^-$

14 e^-, $[Au_{38}(SR)_{24}]^0$

Formation:

- $[Au_{33}(SR)_{22}]^- + [Au_5(SR)_5]^0 + CO + 4\ OH^- \rightarrow [Au_{38}(SR)_{24}]^0 + CO_3^{2-} + 2\ H_2O + 3\ SR^-$
- $[Au_{37}(SR)_{23}]^0 + [Au(SR)_2]^- \rightarrow [Au_{38}(SR)_{24}]^0 + SR^-$

Consumption:

- $[Au_{38}(SR)_{24}]^0 + [Au_2(SR)_3]^- \rightarrow [Au_{40}(SR)_{25}]^+ + 2\ SR^-$
- $[Au_{38}(SR)_{24}]^0 + [Au_4(SR)_4]^0 + CO + 4\ OH^- \rightarrow [Au_{42}(SR)_{25}]^+ + CO_3^{2-} + 2\ H_2O + 3\ SR^-$
- $[Au_{38}(SR)_{24}]^0 + [Au_4(SR)_5]^- + CO + 4\ OH^- \rightarrow [Au_{42}(SR)_{25}]^+ + CO_3^{2-} + 2\ H_2O + 4\ SR^-$

14 e^-, $[Au_{40}(SR)_{25}]^+$

Formation:

- $[Au_{53}(SR)_{41}]^0 + [Au(SR)_2]^- + [Au_4(SR)_4]^0 + 2\ CO + 8\ OH^- \rightarrow [Au_{40}(SR)_{25}]^+ + [Au_{18}(SR)_{14}]^{2+} + 2\ CO_3^{2-} + 4\ H_2O + 8\ SR^-$
- $[Au_{53}(SR)_{41}]^0 + [Au(SR)_2]^- + [Au_4(SR)_5]^- + 2\ CO + 8\ OH^- \rightarrow [Au_{40}(SR)_{25}]^+ + [Au_{18}(SR)_{14}]^{2+} + 2\ CO_3^{2-} + 4\ H_2O + 9\ SR^-$
- $[Au_{38}(SR)_{24}]^0 + [Au_2(SR)_3]^- \rightarrow [Au_{40}(SR)_{25}]^+ + 2\ SR^-$

Consumption:

- $[Au_{40}(SR)_{25}]^+ + [Au_{11}(SR)_{11}]^0 + 2\ SR^- \rightarrow [Au_{51}(SR)_{38}]^-$
- $[Au_{40}(SR)_{25}]^+ + [Au_2(SR)_3]^- + CO + 4\ OH^- \rightarrow [Au_{42}(SR)_{25}]^+ + CO_3^{2-} + 2\ H_2O + 3\ SR^-$

14 e^-, $[Au_{51}(SR)_{38}]^-$

Formation:

- $[Au_{53}(SR)_{41}]^0$ + CO + 4 OH^- → $[Au_{51}(SR)_{38}]^-$ + $[Au_2(SR)_3]^-$ + CO_3^{2-} + 2 H_2O
- $[Au_{40}(SR)_{25}]^+$ + $[Au_{11}(SR)_{11}]^0$ + 2 SR^- → $[Au_{51}(SR)_{38}]^-$

Consumption:

- $[Au_{51}(SR)_{38}]^-$ + CO + 4 OH^- → $[Au_{42}(SR)_{25}]^+$ + $[Au_9(SR)_9]^0$ + CO_3^{2-} + 2 H_2O + 4 SR^-

16 e^-, $[Au_{42}(SR)_{25}]^+$

Formation:

- $[Au_{38}(SR)_{24}]^0 + [Au_4(SR)_4]^0 + CO + 4\ OH^- \rightarrow [Au_{42}(SR)_{25}]^+ + CO_3^{2-} + 2\ H_2O + 3\ SR^-$
- $[Au_{38}(SR)_{24}]^0 + [Au_4(SR)_5]^- + CO + 4\ OH^- \rightarrow [Au_{42}(SR)_{25}]^+ + CO_3^{2-} + 2\ H_2O + 4\ SR^-$
- $[Au_{40}(SR)_{25}]^+ + [Au_2(SR)_3]^- + CO + 4\ OH^- \rightarrow [Au_{42}(SR)_{25}]^+ + CO_3^{2-} + 2\ H_2O + 3\ SR^-$
- $[Au_{51}(SR)_{38}]^- + CO + 4\ OH^- \rightarrow [Au_{42}(SR)_{25}]^+ + [Au_9(SR)_9]^0 + CO_3^{2-} + 2\ H_2O + 4\ SR^-$
- $2\ [Au_{43}(SR)_{24}]^+ + 3\ SR^- \rightarrow [Au_{44}(SR)_{26}]^{2-} + [Au_{42}(SR)_{25}]^+$

Consumption:

- $[Au_{42}(SR)_{25}]^+ + [Au(SR)_2]^- + CO + 4\ OH^- \rightarrow [Au_{43}(SR)_{24}]^+ + CO_3^{2-} + 2\ H_2O + 3\ SR^-$

18 e^-, $[Au_{43}(SR)_{24}]^+$

Formation:

- $[Au_{42}(SR)_{25}]^+ + [Au(SR)_2]^- + CO + 4\ OH^- \rightarrow [Au_{43}(SR)_{24}]^+ + CO_3^{2-} + 2\ H_2O + 3\ SR^-$
- $[Au_{33}(SR)_{22}]^- + [Au_{20}(SR)_{14}]^0 \rightarrow [Au_{43}(SR)_{24}]^+ + [Au_{10}(SR)_{10}]^0 + 2\ SR^-$

Consumption:

- $[Au_{43}(SR)_{24}]^+ + [Au(SR)_2]^- + CO + 4\ OH^- \rightarrow [Au_{44}(SR)_{26}]^{2-} + CO_3^{2-} + 2\ H_2O$
- $[Au_{43}(SR)_{24}]^+ + [Au_4(SR)_5]^- + CO + 4\ OH^- \rightarrow [Au_{46}(SR)_{27}]^- + [Au(SR)_2]^- + CO_3^{2-} + 2\ H_2O$

20 e^-, $[Au_{44}(SR)_{26}]^{2-}$

Formation:

- $[Au_{43}(SR)_{24}]^+ + [Au(SR)_2]^- + CO + 4\ OH^- \rightarrow [Au_{44}(SR)_{26}]^{2-} + CO_3^{2-} + 2\ H_2O$
- $[Au_{46}(SR)_{27}]^- + 2\ SR^- \rightarrow [Au_{44}(SR)_{26}]^{2-} + [Au_2(SR)_3]^-$

20 e^-, $[Au_{46}(SR)_{27}]^-$

Formation:

- $[Au_{43}(SR)_{24}]^+ + [Au_4(SR)_5]^- + CO + 4\ OH^- \rightarrow [Au_{46}(SR)_{27}]^- + [Au(SR)_2]^- + CO_3^{2-} + 2\ H_2O$

Consumption:

- $[Au_{46}(SR)_{27}]^- + 2\ SR^- \rightarrow [Au_{44}(SR)_{26}]^{2-} + [Au_2(SR)_3]^-$

Supplementary References

1. Luo, Z., *et al.* Toward understanding the growth mechanism: Tracing all stable intermediate species from reduction of Au(I)–thiolate complexes to evolution of Au_{25} nanoclusters. *J. Am. Chem. Soc.* **136**, 10577-10580 (2014).

2. Perdew, J. P., Burke, K. & Ernzerhof, M. Generalized gradient approximation made simple. *Phys. Rev. Lett.* **77**, 3865-3868 (1996).

3. Ahlrichs, R., Bär, M., Häser, M., Horn, H. & Kölmel, C. Electronic structure calculations on workstation computers: The program system turbomole. *Chem. Phys. Lett.* **162**, 165-169 (1989).

4. Andrae, D., Häußermann, U., Dolg, M., Stoll, H. & Preuß, H. Energy-adjustedab initio pseudopotentials for the second and third row transition elements. *Theor. Chim. Acta.* **77**, 123-141 (1990).

5. Klamt, A. & Schuurmann, G. COSMO: A new approach to dielectric screening in solvents with explicit expressions for the screening energy and its gradient. *J. Chem. Soc., Perkin Trans. 2*, 799-805 (1993).
